# Strong, tough, ionic conductive, and freezing-tolerant all-natural hydrogel enabled by cellulose-bentonite coordination interactions

**DOI:** 10.1038/s41467-022-30224-8

**Published:** 2022-06-21

**Authors:** Siheng Wang, Le Yu, Shanshan Wang, Lei Zhang, Lu Chen, Xu Xu, Zhanqian Song, He Liu, Chaoji Chen

**Affiliations:** 1 Jiangsu Key Laboratory of Biomass Energy and Material, Institute of Chemical Industry of Forestry Products, Chinese Academy of Forestry, 210042 Nanjing, China; 2 Hubei Biomass-Resource Chemistry and Environmental Biotechnology Key Laboratory, School of Resource and Environmental Sciences, Wuhan University, 430079 Wuhan, China; 3 Jiangsu Co–Innovation Center of Efficient Processing and Utilization of Forest Resources, College of Chemical Engineering, Nanjing Forestry University, 210037 Nanjing, China

**Keywords:** Gels and hydrogels, Organic-inorganic nanostructures, Polymers

## Abstract

Ionic conductive hydrogels prepared from naturally abundant cellulose are ideal candidates for constructing flexible electronics from the perspective of commercialization and environmental sustainability. However, cellulosic hydrogels featuring both high mechanical strength and ionic conductivity remain extremely challenging to achieve because the ionic charge carriers tend to destroy the hydrogen-bonding network among cellulose. Here we propose a supramolecular engineering strategy to boost the mechanical performance and ionic conductivity of cellulosic hydrogels by incorporating bentonite (BT) via the strong cellulose-BT coordination interaction and the ion regulation capability of the nanoconfined cellulose-BT intercalated nanostructure. A strong (compressive strength up to 3.2 MPa), tough (fracture energy up to 0.45 MJ m^−3^), yet highly ionic conductive and freezing tolerant (high ionic conductivities of 89.9 and 25.8 mS cm^−1^ at 25 and −20 °C, respectively) all-natural cellulose-BT hydrogel is successfully realized. These findings open up new perspectives for the design of cellulosic hydrogels and beyond.

## Abstract (web summary)

Cellulose based ion conductive hydrogels are emerging materials for application in flexible electronics but achieving simultaneously high conductivity and good mechanical properties remains challenging. Here, the authors propose a supramolecular engineering strategy to strengthen cellulosic hydrogel and to improve simultaneously its ionic conductivity and freezing tolerance.

## Introduction

The past decade has witnessed the fast development of conductive hydrogel as they find huge opportunities in flexible electronics, including touch screens, wearable devices, and flexible energy storage devices, to name a few^1^. Typically, a conductive hydrogel is composed of a polymeric network, absorbed water, and electrically/ionically conductive medium, in which the polymeric network plays a vital role in determining the overall performance^2^. To date, synthetic polymers, such as the most widely used polyacrylic acid (PAA) and polyacrylamide (PAAm), remain the preferable polymeric backbones from both research and commercial perspectives since they endow the hydrogel with mechanical flexibility, high water absorption, and good biocompatibility^3–5^. However, these synthetic polymers are mainly prepared from petrochemicals and are of poor biodegradability, which brings serious harm to the ecological environment.

From the perspective of building a sustainable world, inexhaustible and biodegradable cellulose has long been considered as one of the most promising candidates to replace synthetic polymers^6,7^. Up to now, a wide variety of ionic/electrical conductive cellulose hydrogels have been realized with the introduction of conductive polymers^8^, carbon-based nanomaterials^9^, ionic liquids^10^, and inorganic salt^11^ as the conductive medium. Among them, ionic conductive cellulose hydrogels incorporated with inorganic salt ions gained considerable attention in the field of flexible energy storage devices due to their high ionic conductivity and good freezing tolerance^12,13^. However, since the salt ions can easily diffuse into the semicrystalline/crystalline regions of cellulose and weaken the hydrogen-bonding interaction between the cellulose chains, a trade-off between ionic conductivity and mechanical strength was frequently observed^14,15^.

Recently, several works have been devoted to mitigating the detrimental effect of inorganic salt additives on the mechanical performance of cellulose hydrogels, the strategies involved in these works could be divided into two categories, namely introducing a cross-linked second network to form a double-network structure and constructing chemical crosslinks among the cellulose molecular chains^16^. From the preparation process, one can find that these works inevitably involves the use of petrochemical-derived monomers and tedious chemical reaction step, more importantly, there is a general trend that higher cross-linking density will result in a decrease of ionic conductivity^17,18^. Taking the cellulose-based chemically cross-linked hydrogel developed by He et al. as an example^19^, it delivers a significantly improved fracture stress of ~50 kPa, but accompanied by a lowered ionic conductivity of 0.16 mS cm^−1^. So far, it remains a great challenge to develop cellulose hydrogels with combined advantages of high ionic conductivity and mechanical strength.

**Fig. 1:**
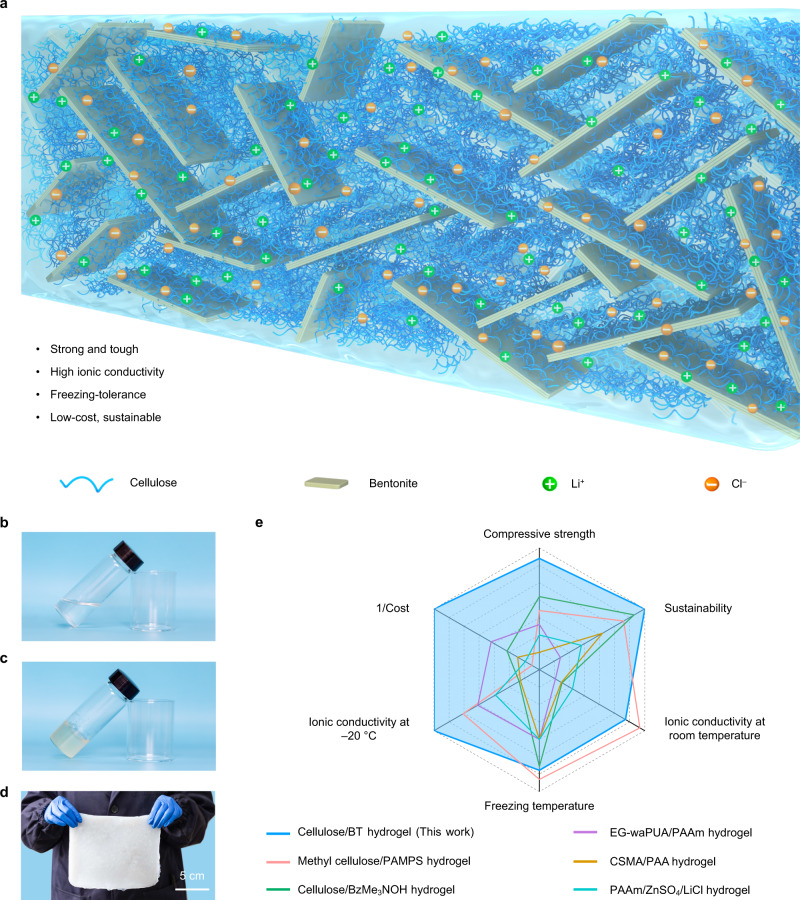
Fabrication of cellulose/BT hydrogels. **a** Schematic of the proposed microstructure for the cellulose/BT hydrogels. **b**, **c** Photographs of the cellulose solution and hydrogel. **d** Optical image of the large-sized Ion-CB hydrogel. **e** A comprehensive comparison between cellulose/BT hydrogel of this work and previously reported conductive hydrogels in terms of compressive strength, sustainability, cost, freezing temperature, and ionic conductivity at room temperature and −20 °C including methyl cellulose/poly (2-acrylamido-2-methylpropanesulfonic acid) (PAMPS) hydrogel^37^, cellulose/benzyltrimethyl ammonium hydroxide (BzMe_3_NOH) hydrogel^38^, ethylene glycol-waterborne anionic polyurethane acrylates (EG-waPUA)/polyacrylamide (PAAm) hydrogel^39^, methacrylated chondroitin sulfate (CSMA)/polyacrylic acid (PAA) hydrogel^40^, and polyacrylamide (PAAm)/ZnSO_4_/LiCl hydrogel^41^.

On the basis of the above discussion, we anticipate that constructing a reasonable and robust bond network within the cellulose hydrogel, rather than blindly increasing the chemical cross-linking density, may be a more feasible approach to improve the mechanical strength without sacrificing ionic conductivity. As a kind of natural clay, bentonite (BT) nanoplatelets have an extremely high in-plane elastic modulus and are rich in exposed bonding sites on its surface, which has been demonstrated to be effective for enhancing the mechanical strength of polymer-based composite materials^20,21^. Inspired by these attractive features, we develop a strong and highly ionic conductive all-natural cellulose-BT hydrogel, in which the BT nanoplatelets promise enhanced mechanical strength and the interstitial space between adjacent BT nanoplatelets serves as straight channels for fast ion transport (Fig. 1a and Supplementary Fig. 1). The introduction of inorganic salts significantly enhances the freezing tolerance of the hydrogel, as a result, advantages of high mechanical strength and ionic conductivity can be largely retained at extremely low temperatures. Combined with the simplicity and scalability of the preparation method, as well as the high natural abundance of cellulose and BT, the cellulose/BT hydrogel developed here holds great potential for practical use in flexible electronics.

## Results

### Fabrication of the cellulose/BT hydrogels

The cellulose/BT mixture solution shown in Fig. 1b appears homogeneous and transparent, suggesting a stable dispersion of BT nanoplatelets in the solution. After 2 h gelation, a non-flowing cellulose/BT hydrogel was obtained and termed as alkaline hydrogel (Fig. 1c). It was then immersed in deionized water to remove NaOH and urea until a pH of around 7.0 was detected, the alkaline hydrogel become neutral as expected, this process was accompanied by an obvious color change from transparent to white (Supplementary Fig. 2). Next, the neutral hydrogel was soaked in LiCl aqueous solution to obtain the ionic conductive cellulose-BT (abbreviated as Ion-CB hereafter, the prefix “Ion-” means the hydrogel is doped with LiCl and ionic conductive) hydrogel (Fig. 1d). The LiCl salt concentration distribution across the Ion-CB hydrogel from the outside surface to the inner part is uniform (Supplementary Fig. 3). Meanwhile, the salt concentration of the Ion-CB hydrogel can be affected by the sample size (i.e., sample with a larger size has a slightly lower salt concentration) and immersing conditions such as the salt concentration of the soaking solution (i.e., the higher salt concentration of the soaking solution, the higher salt concentration of the obtained hydrogel sample), immersion time (i.e., prolonging the immersion time slightly increases the salt concentration), and immersion temperature (i.e., increasing the immersion temperature leads to a slightly increased salt concentration) (Supplementary Fig. 4). Therefore, the salt concentration of the Ion-CB hydrogel can be facilely tuned by adjusting the fabrication conditions as needed. Supplementary Fig. 5 reveals that the stress-strain curves for the cellulose/BT hydrogels with an immersion time of 7 and 120 days are almost overlapped, in sharp contrast, the failure compressive stress of cellulose hydrogel decreased dramatically with increasing immersion time, which accounts for only 5.6% of that of Ion-CB hydrogel after 120 days, implying that the LiCl would not result in structural deterioration when BT nanoplatelets are incorporated. With further consideration of the high abundance and sustainability of raw materials, the Ion-CB hydrogel exhibits an unprecedented combination of high mechanical strength, high ionic conductivity, and low cost, which outperforms the vast majority of ionic conductive hydrogel reported previously (Fig. 1e).

### Characterization of the cellulose-BT interactions

**Fig. 2:**
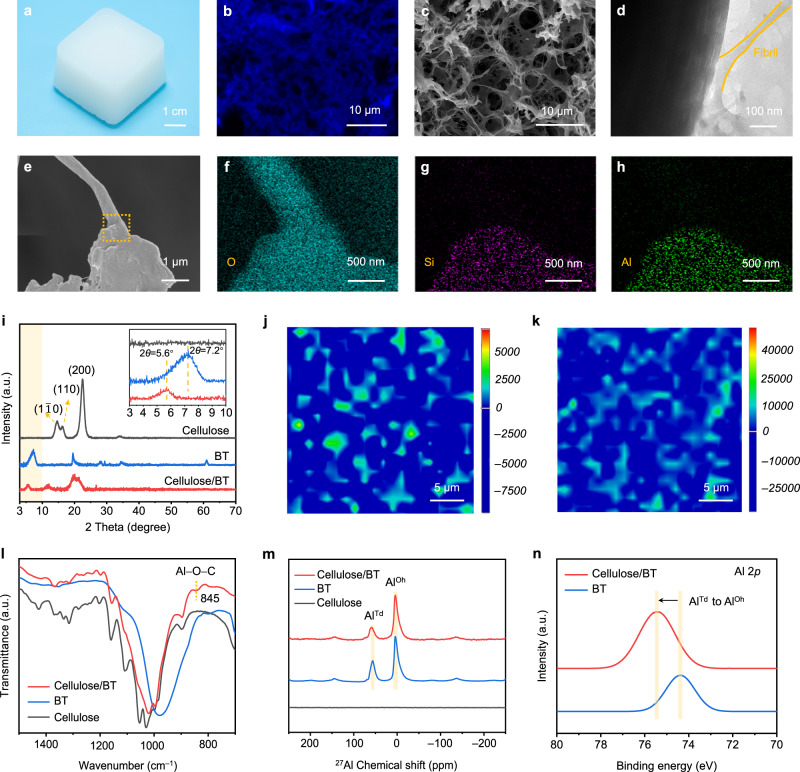
Structure and morphology analysis of cellulose, BT, and cellulose/BT hydrogel. **a** Photograph of the Ion-CB hydrogel. **b** CLSM image of the Ion-CB hydrogel. **c** SEM image of the Ion-CB hydrogel shows the porous network. **d** TEM image of the mixture for cellulose and BT. **e** SEM image of the Ion-CB hydrogel under high magnification to show interactions between cellulose and BT. **f**–**h** EDX mappings of O (**f**), Si (**g**), and Al (**h**) in the Ion-CB hydrogel. **i** XRD patterns of cellulose, BT, and Ion-CB hydrogel. The inset corresponds to their XRD patterns at 2*θ* = 3° to 10°. **j**, **k** Two-dimensional (2D) Raman images of the Ion-CB hydrogel with BDE (**j**) and without BDE (**k**) obtained from the −OH stretching intensities (3000–3400 cm^−1^). Blue corresponds to the chemically cross-linked domains, and green indicates the uncross-linked domains. **l**, **m** FTIR (**l**) and ^27^Al solid-state MAS NMR spectra (**m**) of cellulose, BT, and Ion-CB hydrogels. **n** Al 2*p* orbital XPS spectra of BT and Ion-CB hydrogel.

Figure 2a presents the optical image of a cubic Ion-CB hydrogel with an edge size of 4 cm, which demonstrates good dimensional stability without liquid leakage over a long period of storage time. The confocal laser scanning microscopy (CLSM) and scanning electron microscopy (SEM) images shown in Fig. 2b, c jointly reveal a uniform and 3D connected network in the Ion-CB hydrogel, which is often observed in cellulose gel materials (Supplementary Figs. 6 and 7)^22,23^. The subtle interaction between cellulose and BT nanoplatelets was investigated by transmission electron microscopy (TEM) and energy dispersive spectrometer (EDS) elemental mapping analysis (Fig. 2d–h, and Supplementary Fig. 8), it is observed that the cellulose nanofibrils and BT nanoplatelets are closely entangled with each other, giving visible evidence of strong cellulose-BT interaction. XRD, Raman, and rheology measurements were performed to gain a comprehensive understanding of the cellulose-BT interaction. Figure 2i shows the XRD patterns of cellulose, BT, and Ion-CB hydrogel, the characteristic peak of BT located at 7.2° shifts to 5.6° in the Ion-CB hydrogel, suggesting an increase of the interlayer spacing of BT resulted from cellulose intercalation into BT^24,25^. Figure 2j, k compares the 2D Raman mapping images of Ion-CB hydrogels prepared with and without cross-linker 1,4-Butanediol diglycidyl ether (BDE), in which the blue-colored region indicates higher cross-linking density, while the green-colored region indicates the opposite. It is surprising to find that they deliver similar cross-linking densities, both of which are much higher than that obtained for the ionic conductive cellulose (Ion-C) hydrogel prepared in absence of BT (Supplementary Fig. 9), demonstrating that BT contributes a lot in enabling high cross-linking density. Rheology measurement results provide another solid evidence to support the above view. As shown in Supplementary Fig. 10, for the cellulose/BT solution, a sol-gel phase transition indicated by the observation that G’ became higher than G” occurs within 7 min, demonstrating the formation of a cross-linked network, while such a phase transition was not observed for the cellulose solution without BT.

Fourier transform infrared (FTIR) measurements for the BT, cellulose and the Ion-CB hydrogel were also carried out to provide further experimental evidence to the cellulose-BT interaction. As shown in Fig. 2l, a new characteristic peak at 845 cm^−1^ can be clearly observed in the FTIR spectrum of Ion-CB hydrogel, indicating the formation of Al−O−C bond between cellulose and BT^21,26^. In addition to FTIR, we carried out solid-state ^27^Al spectroscopy to gain further insights into the molecular interactions in the Ion-CB hydrogel (Fig. 2m). The peaks at 57.5 and 3.5 ppm in the ^27^Al MAS NMR spectrum of BT are assigned respectively to tetrahedrally coordinated aluminum (in the lattice cell of [AlO_4_], donated as Al^Td^) and octahedrally coordinated one (in the lattice cell of [AlO_6_], donated as Al^Oh^). Compared to BT, no peak shift or new peak was observed in the spectrum of cellulose/BT (dried Ion-CB), suggesting the coexistence of these two types of lattice Al. Importantly, we notice that the Al^Td^/Al^Oh^ signal intensity ratio presents an obvious decrease in the cellulose/BT, which indicates the partial transformation of Al^Td^ into Al^Oh^ as a result of cellulose-BT coordination interaction. This is in good agreement with the XPS results. As shown in Fig. 2n and Supplementary Fig. 11, the peaks localized at around 74.4 and 75.2 eV correspond to the Al^Td^ and Al^Oh^ states, respectively^27,28^. The binding energy shift further confirms that a substantial of Al^Td^ on the BT surface has transformed into Al^Oh^. All the above findings jointly demonstrate that the Al^Td^ on the BT surface is easily coordinated with O from the cellulose to become Al^Oh^ state, which is responsible for the formation of Al−O−C between BT and cellulose. For the MAS NMR spectra of ^13^C of the cellulose sample, the characteristic peaks located at 71.4 and 74.9 ppm are associated with the C-2, C-3, and C-5, peak at 88.9 ppm belongs to C-4, and peak at 104.3 ppm corresponds to the C-1 (Supplementary Fig. 12). For the Ion-CB sample, the decrease of the sharpness of these peaks points to the increase of the amorphous region (or the decrease of crystallinity) of cellulose^29,30^, as also revealed by the XRD results. No obvious shift of these peaks or new peaks is observed, indicating the chemical surroundings of C in the cellulose remain unchanged. This result is consistent with the above discussions regarding the mechanism of cellulose-BT interaction.

### Theoretical simulation for evaluating the impact of BT

**Fig. 3:**
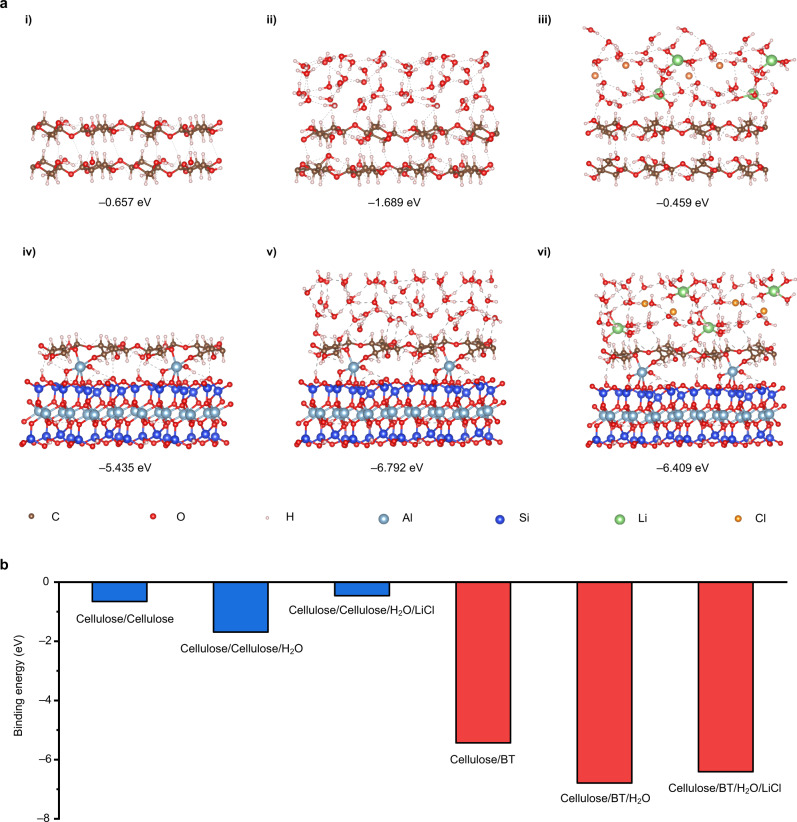
Impact of BT on cellulose-cellulose and cellulose-BT interactions from DFT calculation. **a** Energy-optimized geometry of bonding between cellulose chains by theoretical calculation with DFT study (i–iii). The interaction between two cellulose chains in a solvent-free (i), water environment (ii), and a LiCl/H_2_O solution system (iii). Energy-optimized geometry of bonding between cellulose and BT nanoplatelets via Al substitution sites obtained by theoretical calculation with DFT study (iv–vi). The interaction between cellulose and BT nanoplatelets in a solvent-free (iv), water environment (v), and a LiCl/H_2_O solution system (vi). **b** The binding energy of cellulose and cellulose/BT in different environments by DFT calculation.

The binding energy of intermolecular interaction under different environments was calculated by density functional theory (DFT) with the purpose of gaining further insight into the cellulose-BT interaction. As shown in Fig. 3a(i–iii), the interaction energy within cellulose is significantly lowered to −0.459 eV when LiCl is introduced in the cellulose-water system. This result predicts the poor mechanical strength of ionic conductive cellulose hydrogels incorporated with inorganic salts, which has been proven in this and previous works^31^. The binding energy between cellulose and BT is calculated to be −5.435 eV, which is ascribed to that cellulose and BT are cross-linked through the formation of Al−O−C bond (Fig. 3a(iv–vi)). Compared with the cellulose-BT-water system, even though the introduction of LiCl leads to a slight decrease of binding energy from the initial value of −6.792 to −6.409 eV, it remains one order of magnitude higher than that obtained for the control sample without BT (Fig. 3b). In addition, our simulation results show that, compared to H_2_O, BT is more easily prone to interact with cellulose to form Al−O−C bond, and this bond still maintains a high binding energy even in acidic or alkaline environment (Supplementary Figs. 13 and 14). Based on the above experimental and theoretical simulation results, we conclude that the Al−O−C cross-linkages between cellulose and BT are largely responsible for the excellent mechanical properties of Ion-CB hydrogel.

### Characterization of mechanical performances

**Fig. 4:**
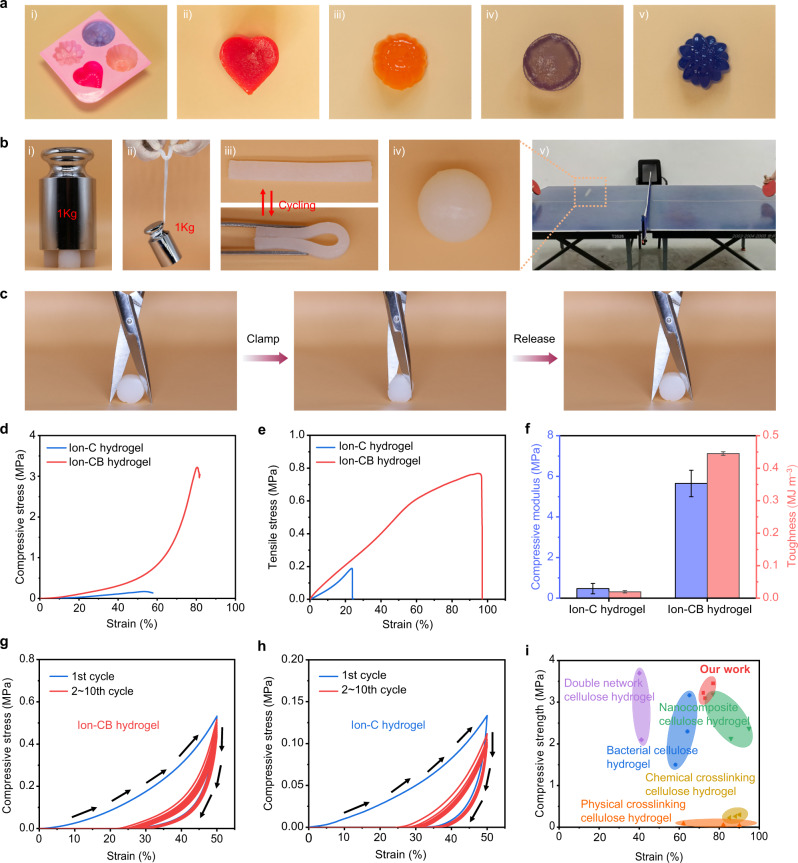
Mechanical properties of the cellulosic hydrogels. **a** Photographs of Ion-CB hydrogels formed in the different mold. **b** Photographs of Ion-CB hydrogels under various external forces. **c** Photographs of the Ion-CB hydrogel cut by a scissor. **d** Compressive stress-strain curves of the hydrogels. **e** Tensile stress-strain curves of the hydrogels. **f** Compressive modulus and toughness of the hydrogels. Data are presented as mean values ± SD, *n* = 3 independent samples. **g**, **h** Cyclic compressive stress-strain curves of Ion-CB hydrogel (**g**) and Ion-C hydrogel (**h**) with a maximum strain of 50%. **i** The mechanical properties of Ion-CB hydrogels and other reported cellulose-based hydrogels, including double network cellulose hydrogel^42^, nanocomposite cellulose hydrogel^43^, chemically cross-linking cellulose hydrogel^44^, physical cross-linking cellulose hydrogel^45^, and bacterial cellulose hydrogel^46^.

As shown in Fig. 4a(i–v), benefiting from the simple preparation process, Ion-CB hydrogels with complicated shapes can be easily prepared, indicating it holds great potential to fit various application scenarios. Figure 4b(i–ii) shows that the Ion-CB hydrogel can easily hold a 1 kg weight with minor deformation, evidencing its high stiffness and toughness. Cyclic bending and impacting tests reveal the Ion-CB hydrogel can recover its initial shape immediately after removing the external force, which is an indicator of good resilience (Fig. 4b(iii–v)). Figure 4c shows the pictures of the cutting deformation test, it is surprising to find the Ion-CB hydrogel can bear very high local stress concentration without visible damage and irreversible deformation, which further demonstrates its high toughness and good resilience (Supplementary Fig. 15 and Supplementary Movie 1). This mechanical characterization method is rarely seen in previous reports, which provides more impressive results to demonstrate the mechanical properties than the conventional strain-stress test alone.

Compressive and tensile stress-strain tests were performed to quantitatively examine the mechanical properties of hydrogel samples. As shown in Fig. 4d, the Ion-CB hydrogel exhibits a maximum compression stress of 3.2 MPa at a fracture strain of 80%, the maximum compression stress and fracture strain are respectively 18.9 times and 1.5 times that of the Ion-C hydrogel. Compared with the cellulose/BT hydrogel and cellulose hydrogel (Supplementary Fig. 16), we found that, for cellulose hydrogel, the introduction of LiCl will lead to a further decrease in maximum compression stress from 0.65 to 0.17 MPa, while for the cellulose/BT hydrogel, its excellent mechanical properties are well retained in the Ion-CB hydrogel. The tensile stress-strain curves show that the tensile fracture stress of Ion-CB hydrogel reaches 0.76 MPa at a high fracture strain of 96%, corresponding to an impressively high fracture energy of 0.45 MJ m^−3^ (Fig. 4e). Figure 4f compares the compressive modulus and fracture energy of the Ion-C hydrogel and Ion-CB hydrogel, a 12-times increase in compressive modulus and 23-times increase in fracture energy were achieved for the Ion-CB hydrogel, demonstrating BT can enable a drastic boost in both strength and toughness in cellulose-based hydrogels.

Cyclic compressive tests at a fixed strain of 50% were performed to confirm the effect of the bonding interaction within different hydrogels. As shown in Fig. 4g, h, both Ion-C hydrogel and Ion-CB hydrogel exhibit nonlinear elastic-inelastic behaviors which feature large hysteresis and shape recovery upon unloading in the 1st cycle, indicating energy dissipation during deformation mainly as a result of the rupture of hydrogen bonds. For the Ion-CB hydrogel, each loading curve can reach the peak value of the 1st cycle, which indicates that coordination networks, including the as-formed Al−O−C, are well retained during repeated deformation tests. In addition, nearly overlapped curves are observed from the 2nd to 10th cycles, which reveals a good strain memory effect. In contrast, the maximum stress of the Ion-C hydrogel decreases continuously upon cycling, which is most likely resulted from the destruction of the hydrogen bonds in cellulose (Supplementary Figs. 17 and 18). These interesting findings further confirm the coordination interactions between cellulose and BT, mainly in the form of Al−O−C bond, are robust enough to ensure excellent mechanical properties. Therefore, the Ion-CB hydrogel developed in this work possesses excellent mechanical properties, which are superior to most existing cellulose-based hydrogels, especially in terms of the compressive strain and fracture stress (Fig. 4i).

We further investigated the reswelling behavior of the Ion-CB hydrogel by freeze-drying the hydrogel followed by reswelling treatment. The reswelled freeze-dried Ion-CB hydrogel demonstrates nearly identical mechanical properties to the original hydrogel, suggesting its excellent recovering capability (Supplementary Figs. 19 and 20). The influence of the pH value of the solution was also investigated. The compressive and tensile testing results all indicate that acidic or alkaline treatment leads to a slight decrease of strength, modulus, and toughness (Supplementary Fig. 21). Such a mild decrease in mechanical performance is mainly ascribed to the slight weakening of the cellulose-BT interaction (i.e., Al−O−C bonds), which can be supported by our experimental (Supplementary Fig. 22) and simulation results (Supplementary Figs. 13 and 14).

### Examination and demonstration of high ionic conductivity and freezing tolerance

**Fig. 5:**
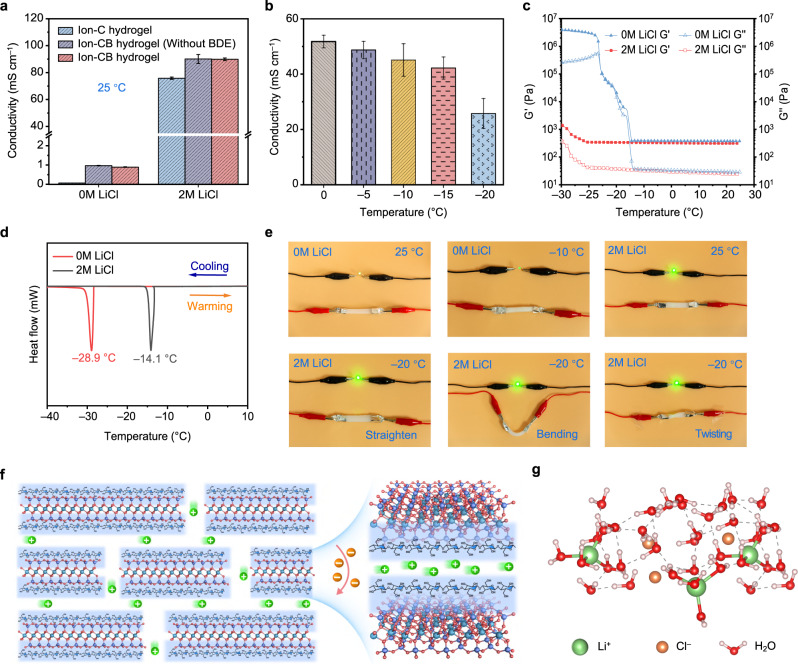
Study of the mechanism of high ionic conductivity and anti-freezing property. **a** Ionic conductivity of hydrogels at 25 °C. **b** Ionic conductivity of Ion-CB hydrogels with varying temperatures. **c**, **d** Storage moduli (G’) and loss moduli (G”) (**c**) and DSC curves (**d**) of cellulose/BT hydrogel and Ion-CB hydrogel. **e** Comparisons of luminance of LEDs (working voltage of 3.0 V) using cellulose/BT hydrogel and Ion-CB hydrogel as the conductor in the flat, bending, and twisting state at varying temperatures. **f** The ions move on the layered BT nanoplatelets with cellulose fibers. **g** Schematic diagram for freezing tolerance mechanism of the hydrogel. Data in (**a**, **b**) are presented as mean values ± SD, *n* = 3 independent samples.

Figure 5a plots the ionic conductivity of three investigated samples obtained by fitting the electrochemical impedance spectroscopy (EIS) spectra to the equivalent circuit (Supplementary Fig. 23). As shown the cellulose hydrogel soaked with 0 M LiCl is non-conductive due to the absence of ion carriers, as expected. An abnormal but interesting phenomenon that the cellulose/BT hydrogel can demonstrate a reasonable ionic conductivity of around 1 mS  cm^−1^ was observed, which indicates that BT changes the ion environment and participate in the ion conduction process within the hydrogel. The Ion-CB hydrogel (cellulose/BT hydrogel soaked with 2 M LiCl) exhibits an impressively high ionic conductivity of 89.9 mS cm^−1^, which is 19 % higher than that of Ion-C hydrogel. A comparison of ionic conductivity for Ion-CB hydrogels with/without BDE reveals the introduction of cross-linker did not lead to reduced ion mobility, suggesting the BT nanoplatelets can improve the ion transport and compensate for the negative effect of high cross-linking density. The fundamental mechanism of how the BT nanoplatelets improve the ionic conductivity will be discussed below. Ionic conductivities of the Ion-CB hydrogel at subzero temperatures were characterized and shown in Fig. 5b and Supplementary Fig. 24, a natural trend that ionic conductivity decreases with the decrease of temperature was observed. When the temperature drops to −20 °C, the Ion-CB hydrogel can still deliver a high ionic conductivity of 25.8 mS cm^−1^, exceeding most previously reported conductive hydrogels (Supplementary Table 1).

To investigate the influence of LiCl on the phase transition behavior of the cellulose/BT hydrogel, dynamic mechanical analysis (DMA) and differential scanning calorimetry (DSC) measurements were conducted. As shown in Fig. 5c, a sharp increase of both G’ and G” is observed for the cellulose/BT hydrogel when the temperature drops to −14.1 °C, suggesting the occurrence of water crystallization. G’ and G” of the cellulose/BT hydrogel soaked with 2 M LiCl (Ion-CB hydrogel) remain stable when the temperature decreases from 25 to −30 °C, further decrease in temperature only results in a slight increase of G’ and G”, indicating the freezing point of the Ion-CB hydrogel is around −30 °C, which is significantly lower than that of the cellulose/BT hydrogel. This result is in good agreement with the DSC measurement result shown in Fig. 5d, where the peaks located at −14.1 and −28.9 °C correspond to the freezing point of the cellulose/BT hydrogel and the Ion-CB hydrogel, respectively. Similar results are also observed for the hydrogels without BT (Supplementary Fig. 25). Hence, it can be concluded that the introduction of LiCl did restrain the solidification of hydrogels, enabling high ionic conductivity at extremely low temperatures. Figure 5e and Supplementary Movie 2 demonstrate the combined advantages of high ionic conductivity, good mechanical properties, and freezing tolerance of the Ion-CB hydrogel, as shown, a light-emitting diode (LED) can be lighted even when the Ion-CB hydrogel connector is bent and twisted at −20 °C (Supplementary Fig. 26). Impressively, the Ion-CB hydrogel still possesses desirable mechanical properties even at an extremely low temperature of −20 °C (Supplementary Fig. 27), which combined with its excellent low-temperature ionic conductive property positions the Ion-CB hydrogel as a competitive material for ionic and biomedical applications.

In the end, we propose a functioning mechanism to explain how cellulose, BT, and LiCl work synergistically in the ionic conductive cellulose/BT hydrogel (Fig. 5f, g). First and foremost, the detrimental effect of LiCl on the mechanical properties of cellulose is overwhelmed with the introduction of BT nanoplatelets through the formation of strong cellulose-BT interactions. Secondly, LiCl significantly lowers the freezing point via weakening the internal hydrogen bonding between water molecules, endowing the hydrogel with high ionic conductivity even at subzero temperatures. Thirdly, according to the zeta potential measurement results, the cellulose/BT nanocomposite is negatively charged, and the interstitial space between adjacent BT nanoplatelets separated by the cellulose can serve as fast cation transport channels, which is considered to further improve the ionic conductivity (Supplementary Fig. 28)^32^. These benefits together contribute to a strong, highly ionic conductive, and freezing-tolerant hydrogel.

Such anti-freezing and conductive hydrogels hold great promise for use in flexible electronics under harsh conditions. As a proof-of-concept demonstration, we applied it as a sensor to detect body movements and physiological signs, human behaviors, including touching, stretching, bending, and swallowing can be monitored by the relative resistance response associated with external force-induced deformation. More importantly, when the hydrogel was adhered to the human model and bent at −20 °C, repeatable resistance responses with a high signal-to-noise ratio are also achieved, implying a long service life and good reliability of the Ion-CB hydrogel (Supplementary Fig. 29 and Supplementary Movie 3).

## Discussion

In this work, we propose an all-natural strategy for the design and construction of mechanically strong and ionic conductive hydrogels from the resource-abundant cellulose and BT in nature. The BT nanoplatelets were demonstrated to enable the formation of dense and strong Al−O−C cross-linkages and hydrogen bond networks in the cellulose/BT hydrogel, fully mitigating the detrimental effects of inorganic salts on the mechanical properties. In addition, BT nanoplatelets separated by the cellulose macromolecules can serve as fast ion transport channels, which further improves the ionic conductivity. Meanwhile, the inorganic salts ensure good freezing tolerance and lead to enhanced mechanical flexibility and ionic conductivity of the hydrogel at low temperatures. As a result, a rationally designed cellulose/BT hydrogel with combined superiorities of excellent mechanical performances (compressive strength up to 3.2 MPa), extremely high ionic conductivity (89.9 mS cm^−1^), and outstanding freezing tolerance (−28.9 °C) was successfully obtained. The high abundance and sustainability of cellulose and BT, facile and scalable preparation process, as well as excellent mechanical and ionic properties, collectively promise the great potential of our developed all-natural hydrogel for practical applications.

## Methods

### Materials

Poplar powder was provided by Qingdao Ruilibo International Trade Co., Ltd. (Qingdao, China). The poplar pulp was used after removing lignin and hemicellulose and complete drying under vacuum at 75 °C for 24 h. Bentonite was supplied by Shenzhen Jintenglong Industrial Co., Ltd. (Shenzhen, China). Sodium chlorite (NaClO_2_, 80%), acetic acid (CH_3_COOH, 36%), sodium hydroxide (NaOH, 96%), and urea (99%) were of analytical grade and purchased from Nanjing Chemical Reagent Co., Ltd. (Nanjing, China). 1,4-Butanediol diglycidyl ether (BDE, 97%) was obtained from Guangdong Weng Jiang Chemical Reagent Co., Ltd. (Shaoguan, China). Deionized water was gained from a laboratory water purifying system (Hitech Sciencetool, Hitech Instruments Co., Ltd.). All of the chemical reagents were used as received unless otherwise specified.

### Extraction of biomass cellulose

The hemicellulose, lignin, and other components in poplar wood powder were removed by soaking in an alkaline solution to obtain biomass cellulose according to the reported literature^33^. Typically, poplar powders (10 g) were dispersed in NaOH (500 mL, 5 wt%) solution under stirring at 70 °C for 4 h, and then washed with deionized (DI) water, followed by ethanol, to remove the residual impurities for getting the raw cellulose fibers. Subsequently, a certain amount of raw cellulose fibers was dispersed in a solution (200 mL) containing NaClO_2_ (2 wt%) and acetic acid (1.5 mL) and stirred at 60 °C for 4 h to gain a white cellulose dispersion. The cellulose pulp was fabricated by drying resultant cellulose dispersion in a vacuum oven after washing with DI water to neutrality.

### Preparation of cellulose/bentonite (cellulose/BT) hydrogels

Cellulose solution (3 wt%) was prepared by dissolving the cellulose pulp (3 g) in an aqueous solution containing 7 wt% NaOH and 12 wt% urea (97 g) at −12 °C according to the previous report^34^. BT nanoplatelets were prepared by a mechanical exfoliation method^26^. A certain amount of BT (0.3 g) and chemical cross-linker BDE (0.75 g) were added dropwise to the cellulose solution (100 g) for homogeneous mixing. The resultant solution was degassed and transferred into cubic, cylindrical, or spherical molds and kept at 60 °C for 2 h to form the gel. The obtained gels were then immersed in DI water for 3 days to prepare the cellulose/BT hydrogels. Finally, the above samples were soaked in 2 M LiCl solutions for 24 h to obtain the ionic conductive cellulose hydrogels. Detailed composition for all investigated samples was listed in Supplementary Table 2.

### Characterization

Confocal laser scanning microscopy (CLSM) (LSM710, Zeiss, Germany) was used to test the pore structure of the hydrogel stained with fluorescent dye solution. Transmission electron microscope (TEM) and field emission scanning electron microscope (FESEM) of BT were conducted using JEM-2100 UHR (JEOL, Japan) and Regulus 8100 (Hitachi, Japan) instruments to observe the microstructure. Scanning electron microscope (SEM) images of cellulose hydrogels were obtained with an environmental scanning electron microscopy (JSM-7600 Fs, JEOL, Japan), and an energy dispersive spectrometer (EDS) was utilized to analyze the elemental composition. The Fourier transform infrared (FTIR) spectra of the cellulose, BT, and dried cellulose hydrogels were recorded in the wavenumber range from 4000 to 500 cm^−1^ using a Fourier transform infrared spectrometer (Nicolet iS50, Thermo Fisher Scientific, USA). Raman spectroscopy and spatial Raman mapping were performed utilizing a Raman imaging microscope (Thermo Scientific DXR2xi, USA). The wavelength of the excitation laser was 532 nm. Raman mappings (scan range 22 μm × 22 μm; depth of scanning, 22  μm) were collected using a condition that the laser power was 8.0 mW, the exposure time was 0.1 s (10 Hz), and the scan time was 200, and the image pixel size was 1.0 μm. The collected spectra were preprocessed by using cosmic-ray removal, noise filtering, and normalization techniques. The peak area method developed by OMINC software was employed for calculating the chemical cross-linking domains. The X-ray diffraction (XRD) patterns of the cellulose, BT, and freeze-dried cellulose hydrogels in the diffraction angle (2*θ*) range from 3° to 70° were recorded using Siemens D5000 X-ray diffractometer (Siemens, Germany) at a scanning speed of 3° min^−1^. X-ray photoelectron scattering (XPS) spectroscopy analysis was used to explore the chemical composition and the element binding energy changes of hydrogels. All the solid-state NMR experiments were performed on a 600 MHz spectrometer equipped with a MAS probe (AVANCE NEO, Bruker, Germany). The ^27^Al and ^13^C MAS NMR spectra of cellulose, BT, and dried cellulose-BT hydrogel were recorded, respectively. The Inductively coupled plasma-optical emission spectrometry (ICP-OES) (OPTIMA7000, PerkinElmer, USA) was used to test the LiCl concentration in the Ion-CB hydrogel.

### DFT calculation

First-principles calculations were carried out using density functional theory (DFT) with generalized gradient approximation (GGA) of Perdew-Burke-Ernzerhof (PBE) implemented in Vienna Ab-Initio Simulation Package (VASP)^35^. The valence electronic states were expanded on the basis of plane waves with the core-valence interaction represented using the projector augmented plane wave (PAW)^36^ approach and a cutoff of 520 eV. To achieve high accuracy, the Brillouin zone integration was sampled with 3 × 3 × 1 k-grid mesh for geometry optimization. The binding energies of cellulose and cellulose, cellulose and BT in an anhydrous environment, aqueous solution, and electrolyte salt solution have been performed via DFT calculations. Taken cellulose and cellulose in electrolyte salt solution for example, the binding energy was calculated as:

1 $$\varDelta E={E}_{{{{\rm{LiCl}}}}-{{{{\rm{H}}}}}_{2}{{{\rm{O}}}}-{{{\rm{Cel}}}}-{{{\rm{Cel}}}}-{{{{\rm{H}}}}}_{2}{{{\rm{O}}}}-{{{\rm{LiCl}}}}}-({E}_{{{{\rm{LiCl}}}}-{{{{\rm{H}}}}}_{2}{{{\rm{O}}}}-{{{\rm{Cel}}}}}+{E}_{{{{\rm{LiCl}}}}-{{{{\rm{H}}}}}_{2}{{{\rm{O}}}}-{{{\rm{Cel}}}}})$$

where ${E}_{{{{\rm{LiCl}}}}-{{{{\rm{H}}}}}_{2}{{{\rm{O}}}}-{{{\rm{Cel}}}}-{{{\rm{Cel}}}}-{{{{\rm{H}}}}}_{2}{{{\rm{O}}}}-{{{\rm{LiCl}}}}}$ is the total energy for two cellulose chains in the electrolyte salt solution, ${E}_{{{{\rm{LiCl}}}}-{{{{\rm{H}}}}}_{2}{{{\rm{O}}}}-{{{\rm{Cel}}}}}$ is one cellulose chain in the electrolyte salt solution.

A hydrolysis reaction was designed for simulating the energy change value when the Al−O−C bond derived from BT and cellulose was replaced partially by H_2_O and formed the Al−OH bond. The energy change could be calculated by the following equation:

2 $$\varDelta E={E}_{{{{\rm{Al}}}}-{{{\rm{OH}}}}}-{E}_{{{{\rm{Al}}}}-{{{\rm{O}}}}-{{{\rm{C}}}}}-{E}_{{{{{\rm{H}}}}}_{2}{{{\rm{O}}}}}$$

where ${E}_{{{{\rm{Al}}}}-{{{\rm{O}}}}-{{{\rm{C}}}}}$ is the energy of BT-Cellulose, ${E}_{{{{\rm{Al}}}}-{{{\rm{OH}}}}}$ is the energy of the hydrolysis product, ${E}_{{{{{\rm{H}}}}}_{2}{{{\rm{O}}}}}$ is the energy of a single water molecule.

### Mechanical measurements

Compressive measurements were performed on cellulose hydrogels using a universal testing machine (UTM6503, Shenzhen SANS Testing Machine Co. Ltd., China), which was equipped with a 5000 N cell. To evaluate the compressive mechanical performance, cylinder-shaped cellulose hydrogels with a diameter of 10 mm and a height of 10 mm were placed on the center of the lower flat plate and compressed by a constant loading rate of test of 2 mm min^−1^. Cyclic tests were performed by conducting subsequent trials immediately after initial loading. Three samples were employed for the test and the average results were reported. In addition, the modulus of cellulose hydrogel could be calculated by the following equation:

3 $$E=\,\frac{\sigma }{\varepsilon }$$

where *σ* is the stress of the initial linear range of the stress-strain curve and ε is the strain corresponding to the selected stress.

The water content of the cellulose hydrogels can be calculated in an equation as follow:

4 $$S( \% )=\frac{{W}_{{{{\rm{wet}}}}}-{W}_{{{{\rm{dry}}}}}}{{W}_{{{{\rm{wet}}}}}}\times 100 \%$$

where *W*_wet_ and *W*_dry_ are the weight of the cellulose hydrogels before and after drying at 90 °C under vacuum.

### Rheological properties

The rheological properties of cellulose/BT solution (without BDE) were studied using the Thermo Fisher Scientific MARS60 rheometer that was equipped with cone-plate geometry (cone angle = 1°, plate diameter = 10 mm) at 25 and 60 °C. Strain sweep experiments were carried out in the strain range from 0.01 to 100% to determine the linear viscoelastic region under the condition of a fixed frequency of 1 Hz. Tine sweep measurements were conducted at angular frequencies ranging from 1 Hz at a strain of 10% under 25 and 60 °C. Storage and loss modulus of hydrogels were obtained using a TA instruments Q850 in the tension mode with a constant frequency of 1 Hz at a cooling rate of 3 °C min^−1^ from 25 to −90 °C. The freezing temperatures of hydrogels were measured using a differential scanning calorimeter (DSC 8000). The DSC experiments were conducted by cooling the samples at a rate of 5 °C min^−1^ from 25 to −45 °C under nitrogen protection.

### Electrochemical properties

The electrochemical properties of the hydrogels were measured using an electrochemical workstation (CHI 660D, Chenhua). The real-time resistance of the hydrogel sensors under compressing, stretching, or bending was recorded by a digital source meter (Keysight 34461A). The data was obtained using the electrochemical AC impendence spectroscopy (IMP) method of all hydrogels with a frequency range from 10^−1^ to 10^5^ Hz and 0.1 V voltage, under a variety of temperatures of 25, 0, −5, −10, −15, −20 °C. Hydrogels were sandwiched between two thin foils for the measurement and the hydrogels were cut into samples of 10 mm in length, 8 mm in width, and 5 mm in thickness. The ionic conductivity of hydrogels was calculated according to the following equation:

5 $$\sigma =\,\frac{L}{RA}$$

where *L* represents the distance between the two probes, *R* represents the electrical resistance of the hydrogels and *A* represents the cross-sectional area of the hydrogels.

## Supplementary information

Supplementary Information

Description of Additional Supplementary Files

Supplementary Movie 1

Supplementary Movie 2

Supplementary Movie 3

## Data Availability

The data that support the findings of this study are available within this paper and or included in the Supplementary Information, and from the corresponding authors upon request. Source data are provided with this paper.

## Source data

Source Data
