# Supplementary material for: Strong, tough, ionic conductive, and freezing-tolerant all-natural hydrogel enabled by cellulose-bentonite coordination interactions
Source: Nat Commun. 2022 Jun 21;13:3408. doi: 10.1038/s41467-022-30224-8 (PMC9213515; doi:10.1038/s41467-022-30224-8)
Supplement: Supplementary file 1 — Supplementary Information [file 41467_2022_30224_MOESM1_ESM.pdf]

Supplementary Information for

Strong, Tough, Ionic Conductive, Freezing-Tolerant All-Natural Hydrogel Enabled by Cellulose-Bentonite Coordination Interactions

Siheng Wang[1,2,3#], Le Yu[2#], Shanshan Wang[3], Lei Zhang[1], Lu Chen[2], Xu Xu[3], Zhanqian Song[1], He Liu[1]*, Chaoji Chen[2]*

[1] Jiangsu Key Laboratory of Biomass Energy and Material, Institute of Chemical Industry of Forestry Products, Chinese Academy of Forestry, 210042 Nanjing, China.

[2] Hubei Biomass-Resource Chemistry and Environmental Biotechnology Key Laboratory, School of Resource and Environmental Sciences, Wuhan University, 430079 Wuhan, China.

[3] Jiangsu Co–Innovation Center of Efficient Processing and Utilization of Forest Resources, College of Chemical Engineering, Nanjing Forestry University, 210037 Nanjing, China.

[#] Equal contributions.

* Corresponding authors:

Chaoji Chen, email: chenchaojili@whu.edu.cn.

He Liu, email: liuhe.caf@gmail.com.

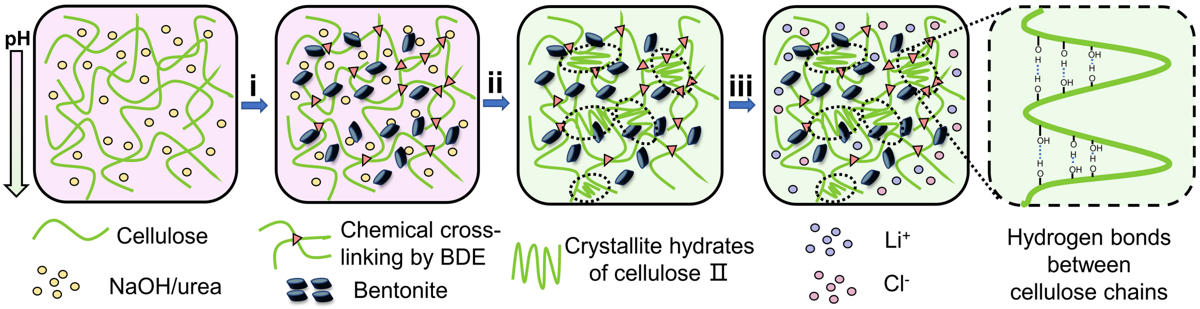

Supplementary Fig. 1 | Schematic illustration of the fabrication process of the cellulose/BT hydrogels. In the process **i**), BT materials were added to cellulose solution to form an alkaline hydrogel under basic conditions. In the process **ii**), the alkaline hydrogels were immersed in deionized water until the pH of the intra-hydrogel environment from basic to acidic conditions, removing excess impurities. In the process **iii**), cellulose/BT hydrogels were immersed in the LiCl solution using a highly adjustable soaking strategy to endow hydrogels with conductivity, as well as freezing tolerance.

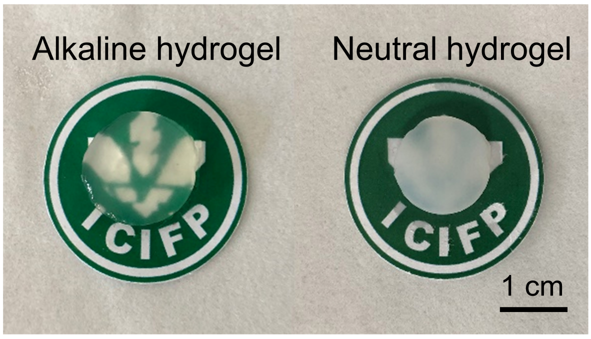

Supplementary Fig. 2 | Photos of (left) alkaline hydrogel and (right) neutral hydrogel after dialysis. Compared to the alkaline hydrogel, the neutral hydrogel exhibited shrinkage and became opaque mainly due to the removal of alkali and urea.

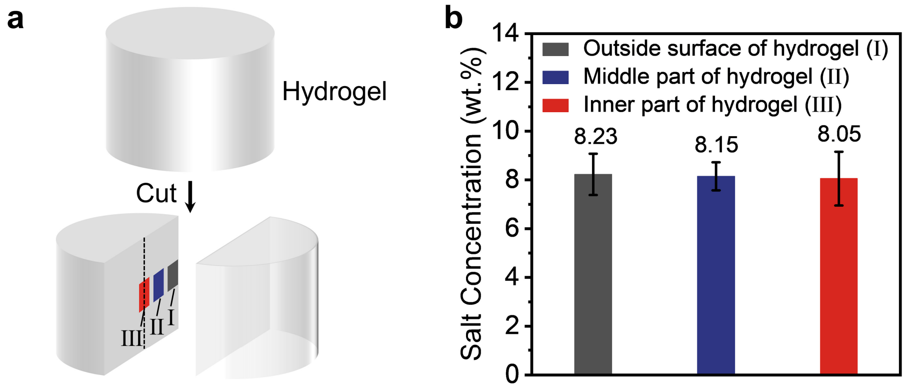

Supplementary Fig. 3 | Experimental measurements to study LiCl salt concentration distribution across the Ion-CB hydrogel. a Schematic diagram of the three parts of the Ion-CB hydrogel used to measure the salt concentration distribution, including the (I) outside surface, (II) middle, and (III) inner parts of the hydrogel. **b** The salt concentration in the outside surface, middle and inner parts of the hydrogel. Data are presented as mean values ± SD, n = 3 independent samples. From the outside surface to the middle and inner parts of the hydrogel, the salt concentrations were 8.23 ± 0.85, 8.15 ± 0.57, and 8.05 ± 1.10 wt.%, respectively, which did not show obvious concentration gradient, indicating that the LiCl was distributed evenly in the hydrogel.

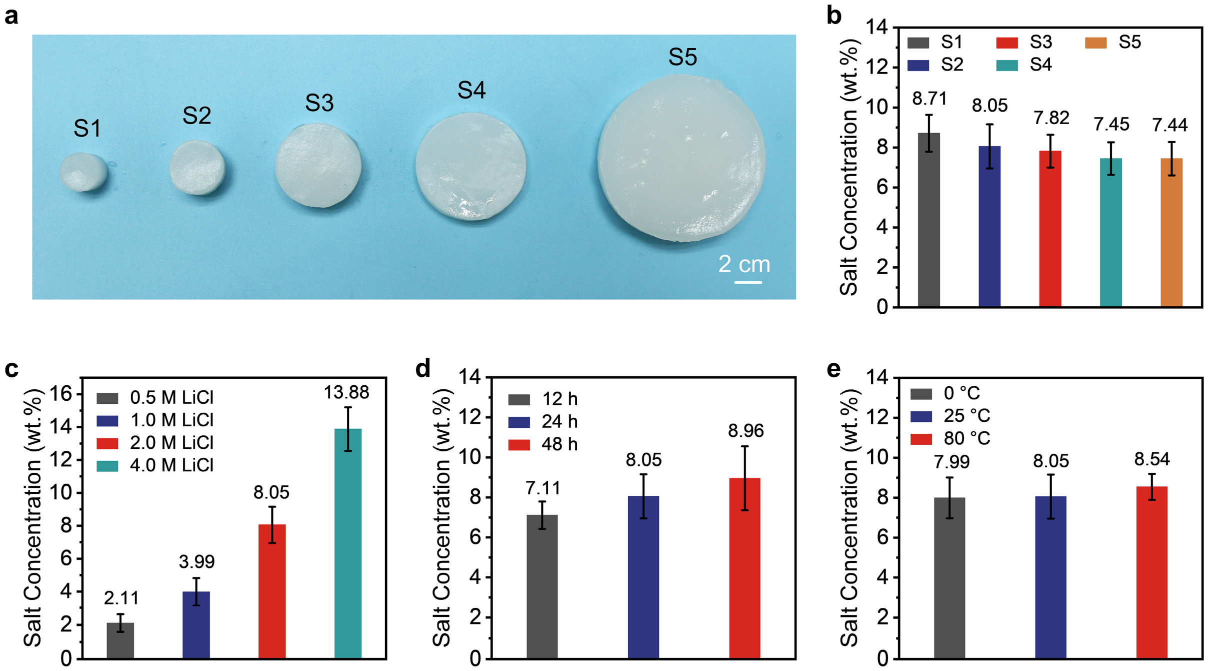

Supplementary Fig. 4 | Experimental measurements to study LiCl salt concentration in the Ion-CB hydrogel under different hydrogel sizes and immersing conditions. a Optical images of five Ion-CB hydrogel samples S1-S5 with different sizes (1.5 cm in height and 1.5, 2, 3, 4, and 6 cm in diameter, respectively). **b** The salt concentration of five Ion-CB hydrogel samples S1-S5 with different sizes. **c-e** The salt concentration of Ion-CB hydrogels obtained with different immersing conditions: (**c**) salt concentration of the soaking solution, (**d**) immersing time; and (**e**) immersing temperature. Data in (**b-e**) are presented as mean values ± SD, *n* = 3 independent samples.

Influence of sample size on the salt concentration distribution. As shown in Supplementary Fig. 4a, we examined the LiCl salt concentration in five cylindrical Ion-CB hydrogel samples with different diameter to explore the relationship between salt concentration and hydrogel size (from hydrogel samples S1 to S5, 1.5 cm in height and 1.5, 2, 3, 4, and 6 cm in diameter, respectively). We found that with the increase of hydrogel size, the salt concentration showed a gradient decrease, and the salt concentration in the hydrogel samples S1 and S5 was 8.71±0.92 and 7.44±0.84 wt.%, respectively (Supplementary Fig. 4b). The maximum difference in salt concentration for the five samples is only 1.27±0.08 wt.%, suggesting that

LiCl has excellent diffusivity in hydrogels, although at larger size. This facilitates the formation of scaled Ion-CB hydrogels.

Influence of immersion conditions on the salt concentration distribution. From Supplementary Fig. 4c-e, the influence of immersing conditions (e.g., solution concentration, immersing temperature, immersing time) on the salt concentration of the hydrogel was further investigated. We found that with the increase of soaking salt concentration, the prolongation of soaking time, and the rise of soaking temperature, the salt concentration in the hydrogel showed a positive correlation trend. Among the three immersing conditions, the salt concentration of the soaking solution shows the most obvious influence on the salt concentration of the final hydrogel product, whereas the immersing temperature has the mildest influence. The above results show that we can facilely tune the salt concentration of the final hydrogel product by controlling the immersing conditions, especially the salt concentration of the soaking solution.

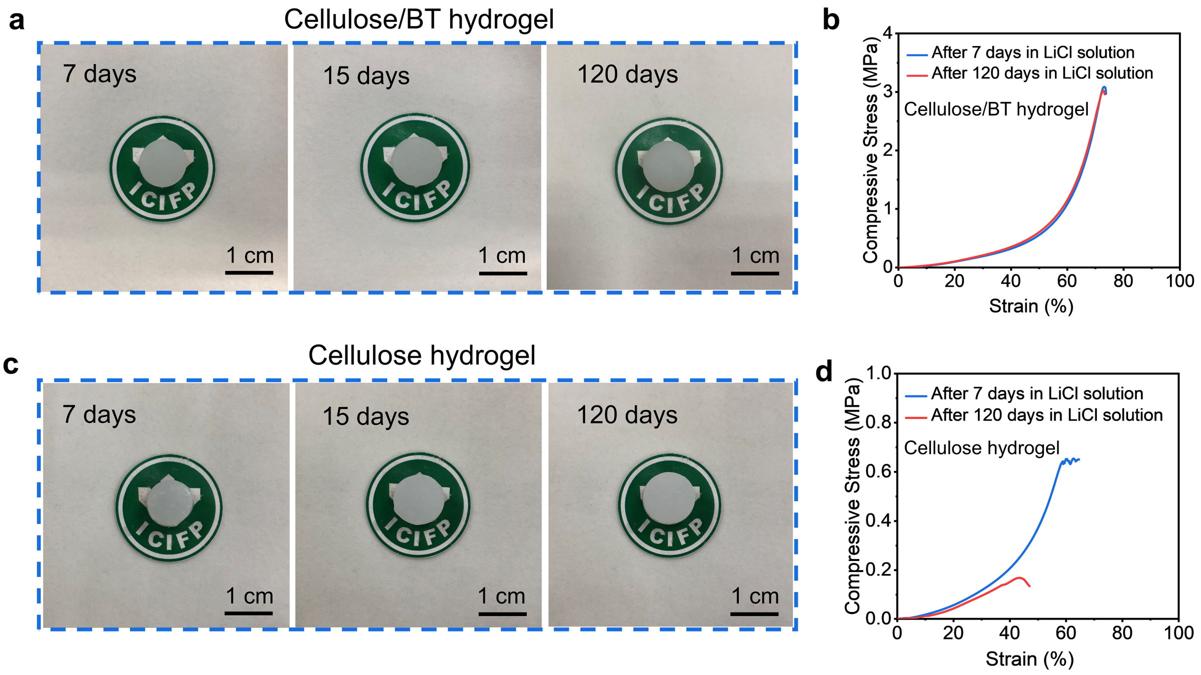

Supplementary Fig. 5 | a Snapshots of cellulose/BT hydrogel sample after 7 days, 15 days, and 120 days soaking in LiCl solution. **b** Compressive stress-strain curves of cellulose/BT hydrogel sample after 7 days and 120 days soaking in LiCl solution. **c** Snapshots of cellulose hydrogel sample after 7 days, 15 days, and 120 days soaking in LiCl solution. **d** Compressive stress-strain curves of cellulose hydrogel sample after 7 days and 120 days soaking in LiCl solution. Compared with the cellulose hydrogel sample, the cellulose/BT hydrogel sample showed no significant difference in volume and mechanical strength after immersing in LiCl solution for 120 days.

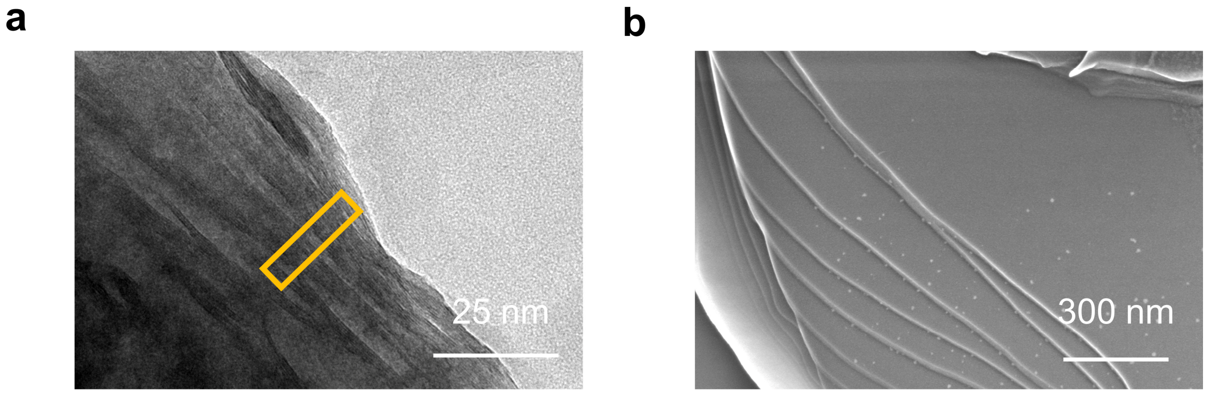

Supplementary Fig. 6 | TEM and SEM images of BT. **a** TEM and **b** SEM images verify the lamellar structure of BT, which is favorable for promoting ion storage and migration.

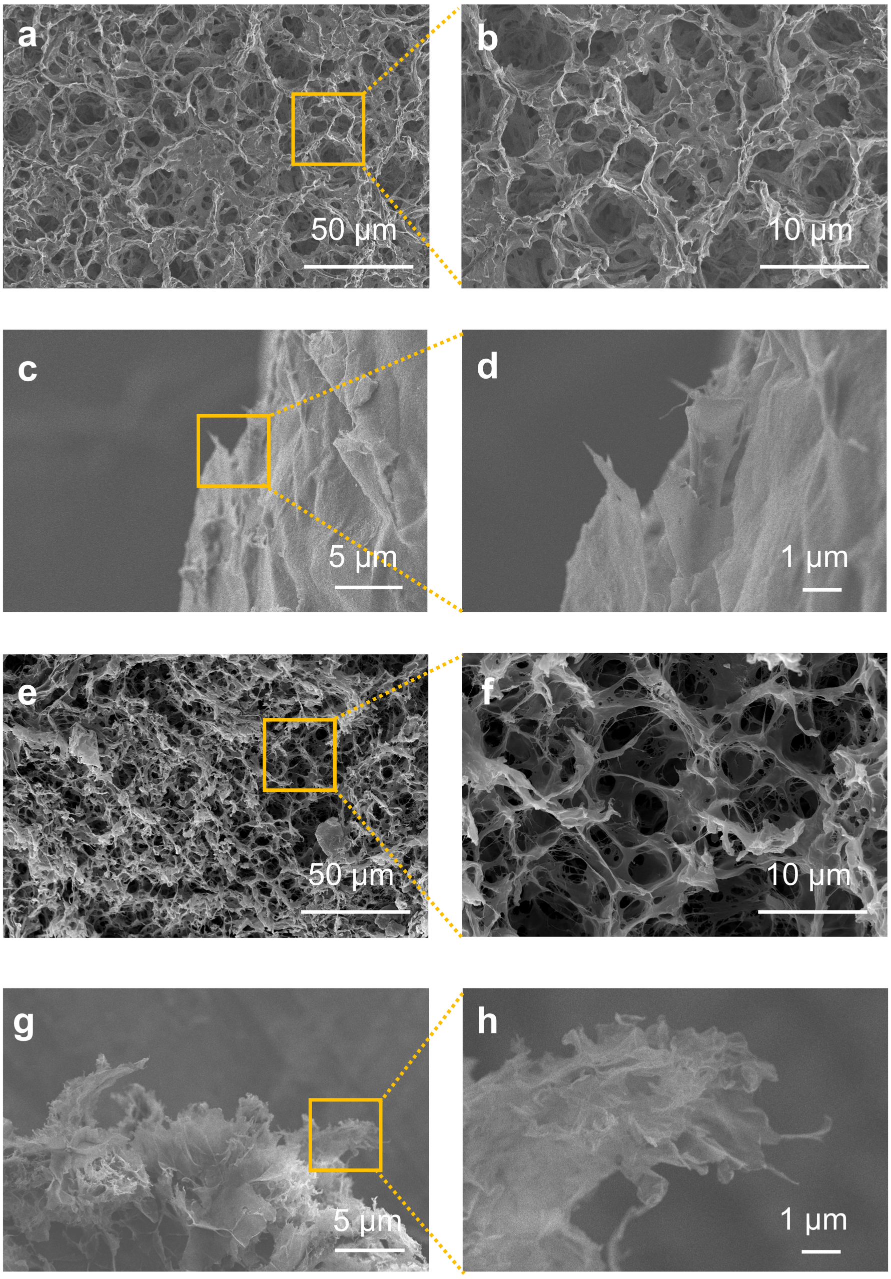

Supplementary Fig. 7 | SEM images of (**a, b**) surface and (**c, d**) cross-section via liquid nitrogen quenching treatment from Ion-C hydrogel samples. SEM images of (**e, f**) surface and (**g, h**) cross-section via liquid nitrogen quenching treatment from Ion-CB hydrogel sample. Compared to the surface morphology of the hydrogel without BT, the surface pore structure of the hydrogel sample decreased significantly after introducing BT, and the pore size became smaller. In addition, due to the intercalation structure formed between BT and cellulose, many fiber filaments protrude from the fracture surface.

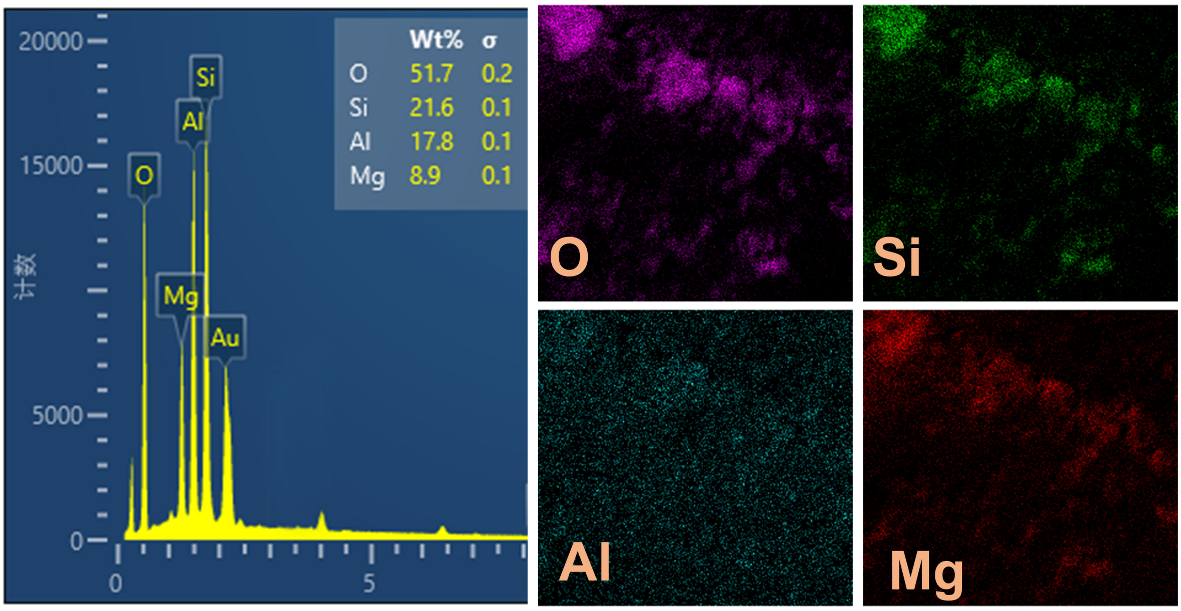

Supplementary Fig. 8 | The element distribution and EDX mappings of O, Si, Al, and Mg in the BT nanoplatelets. The EDX spectrum of BT nanoplatelets shows that it mainly contains O, Si, Al, and Mg elements.

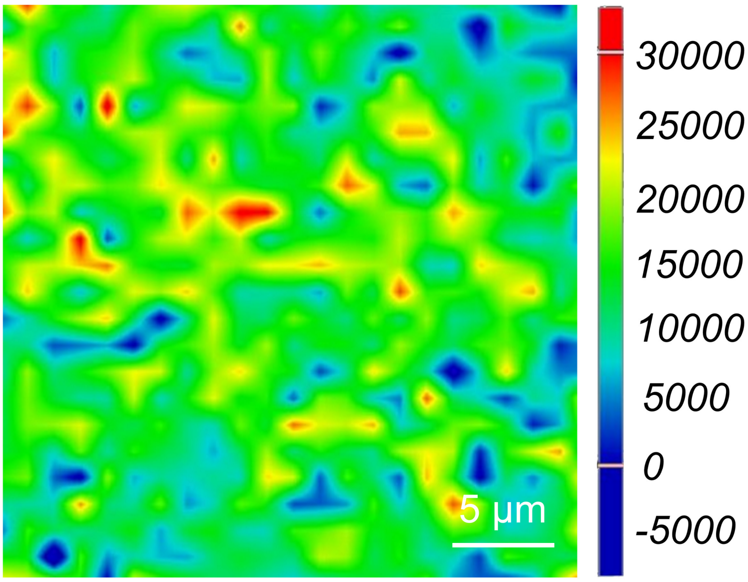

Supplementary Fig. 9 | Two-dimensional (2D) Raman image of pure cellulose hydrogel without BDE and BT obtained from the –OH stretching intensities (3000~3400 cm^{-1}). Blue corresponds to the chemically cross-linked domains, and green indicates the uncross-linked domains. The respective intensities of the –OH stretching at a range of 3000~3400 cm^{-1} in green and blue colors, and the green regions in the 2D images represented the unreacted hydroxyl of cellulose or BT materials, while the blue regions could be attributed to the cross-linked domains. Pure cellulose hydrogel without BDE and BT has a low cross-linking density.

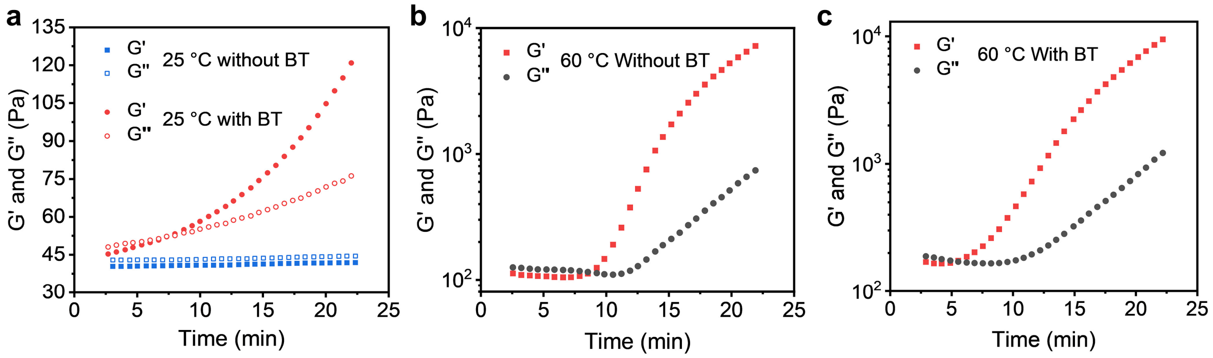

Supplementary Fig. 10 | Rheology measurement. a Elastic (G') and viscous (G'') moduli of the cellulose solution dissolved in alkali urea within and without BT at 25 °C. **b, c** Elastic (G') and viscous (G'') moduli of the cellulose solution dissolved in alkali urea (**b)** without BT and (**c**) containing 10% BT (relative to the mass of cellulose) at 60 °C. A sol-gel transition phenomenon occurs at 25 °C in the cellulose/BT solution within 7 min, proving the formation of a cross-linked network. Moreover, compared to the hydrogel without BT, the gelling speed of the hydrogel containing BT is faster at 60 °C, indicating that BT can promote the formation of the hydrogel.

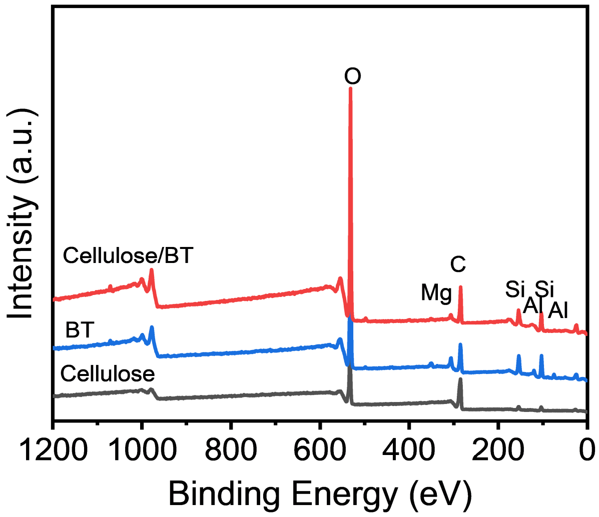

Supplementary Fig. 11 | XPS spectra of the cellulose, BT, and Ion-CB hydrogel. The XPS spectrum of the Ion-CB hydrogel showed that BT was successfully introduced into the nanocomposite hydrogel.

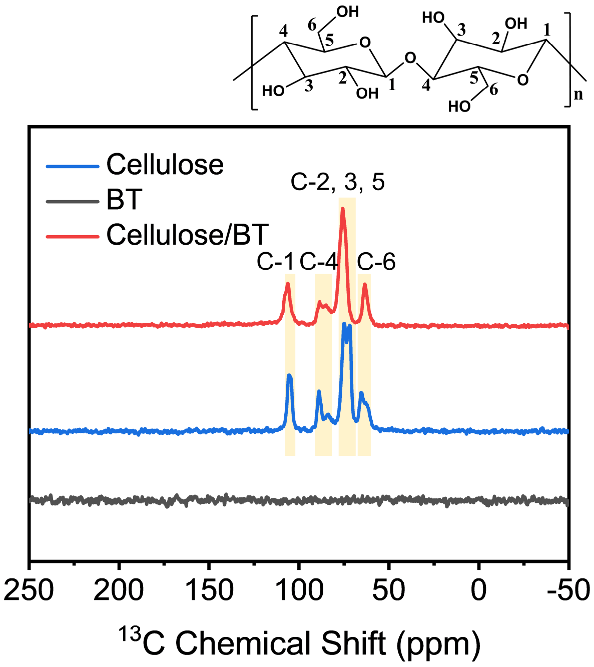

Supplementary Fig. 12 | ^{13}C solid-state MAS NMR spectra of the cellulose, BT, and Cellulose/BT.

For the MAS NMR spectra of ^{13}C of the cellulose sample, the characteristic peaks located at 71.4 and 74.9 ppm are associated with the C-2, C-3, and C-5, peak at 88.9 ppm belongs to C-4, and peak at 104.3 ppm corresponds to the C-1. For the Ion-CB sample, the decrease of the sharpness of these peaks points to the increase of the amorphous region (or the decrease of crystallinity) of cellulose[1, 2], as also revealed by the XRD results. No obvious shift of these peaks or new peaks is observed, indicating the chemical surroundings of C in the cellulose remain unchanged.

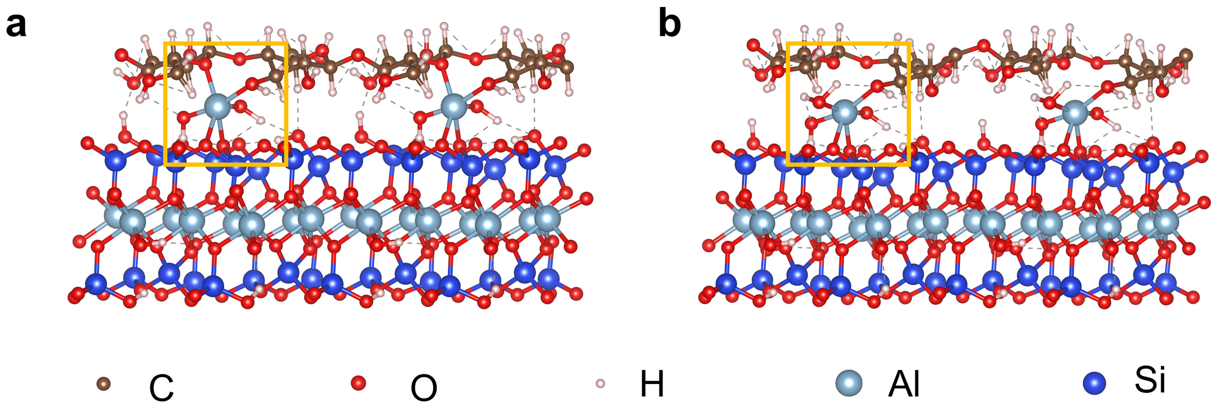

Supplementary Fig. 13 | Impact of H_2O on the cellulose-BT interactions from DFT calculation. a Energy-optimized geometry of bonding between BT nanoplatelets and cellulose obtained by theoretical calculation with DFT study. **b** When BT nanoplatelets are partially combined with water, energy-optimized geometry of bonding between BT and cellulose obtained by theoretical calculation with DFT study.

As shown in Supplementary Fig. 13a, the firm binding between BT and cellulose depends on the formation of the Al–O–C bond, indicating a strong interaction from BT-cellulose system. However, when the Al–O–C bond is substituted partly by H_2O and convert to the Al–OH bond, the energy change value calculated by our simulation in this process is 2.445 eV, indicating that this process is an endothermic reaction (Supplementary Fig. 13b). From the thermodynamic perspective, the binding of BT to water is unfavorable in the system, so BT is more easily prone to form the Al–O–C bond combined with cellulose rather than obtaining the Al–OH bond with H_2O. These results suggest that BT is inclined to form steady Al–O–C bond with cellulose in the hydrogel.

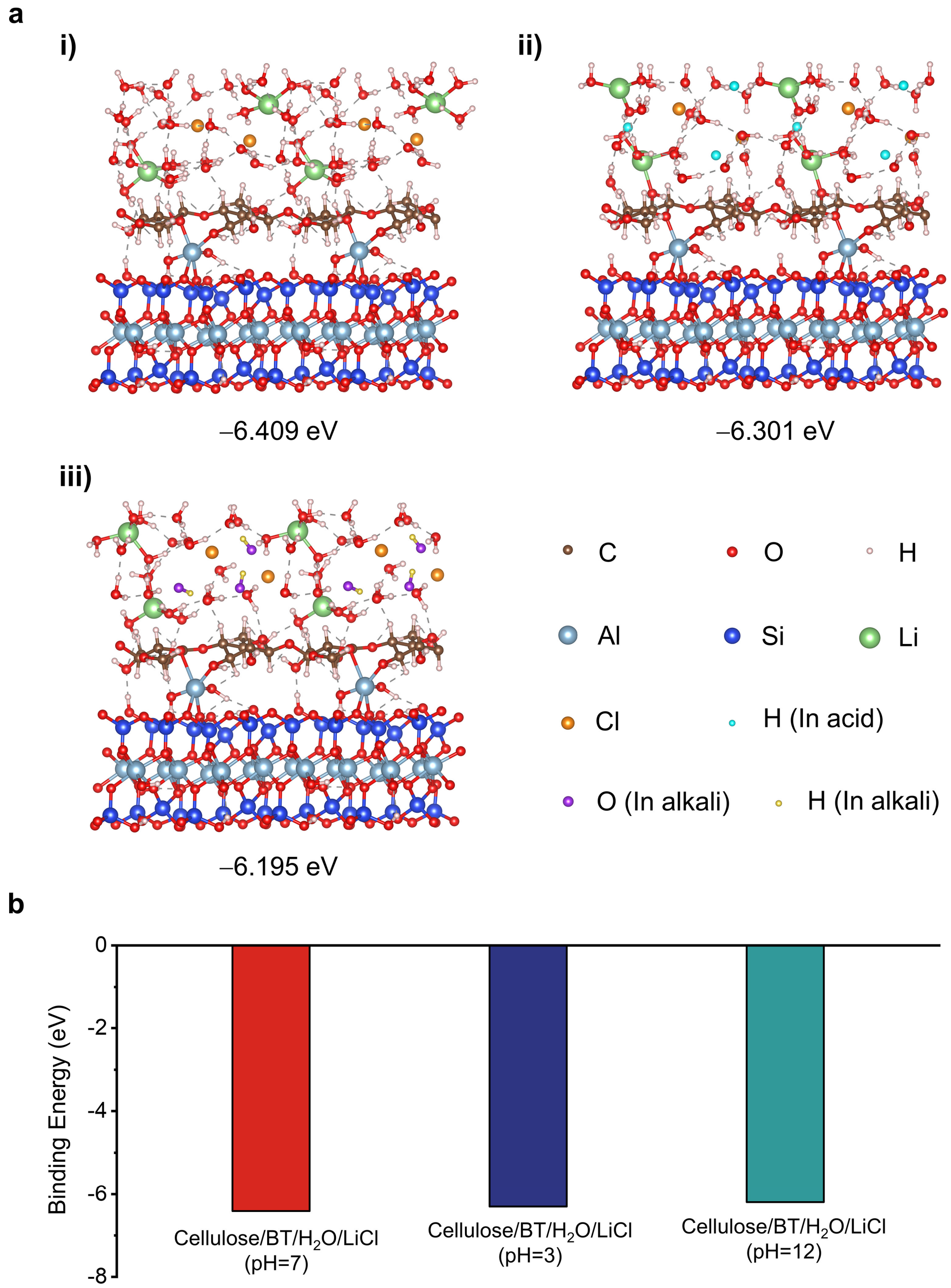

Supplementary Fig. 14 | Impact of pH conditions on the cellulose-BT interactions from DFT calculation. a Energy-optimized geometry of the bonding between cellulose and BT nanoplatelets via Al substitution sites obtained by theoretical calculation with DFT study. (**i-iii**) The interaction between cellulose and BT nanoplatelets at pH value of 7, 3, and 12, respectively. **b** The binding energy of cellulose/BT at varied pH environments by DFT calculation.

As shown in Supplementary Fig. 14a, in a neutral LiCl solution, the binding energy between cellulose and BT is calculated to be −6.409 eV. The addition of acid or alkali to the LiCl solution only results in a slight drop of binding energy from the initial value to −6.301

and −6.195 eV, respectively, both of which are still negative (Supplementary Fig. 14b). The simulation results suggest that the changing of environment from neutral to acidic or alkaline does not deconstruct the formed Al–O–C bond but only slightly reduce the strength of this interaction.

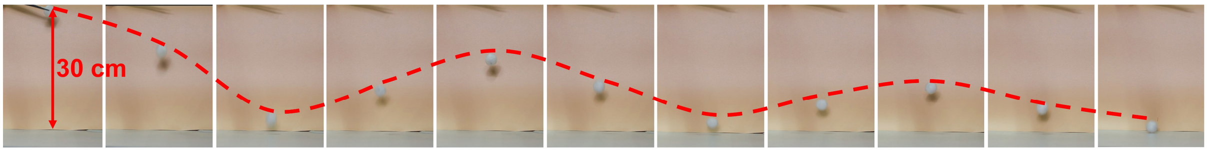

Supplementary Fig. 15 | Rebounding experiments of the Ion-CB hydrogel sample. The Ion-CB hydrogel sample can rebound back and forth without damage when falling in the air, indicating that it has high toughness and elasticity.

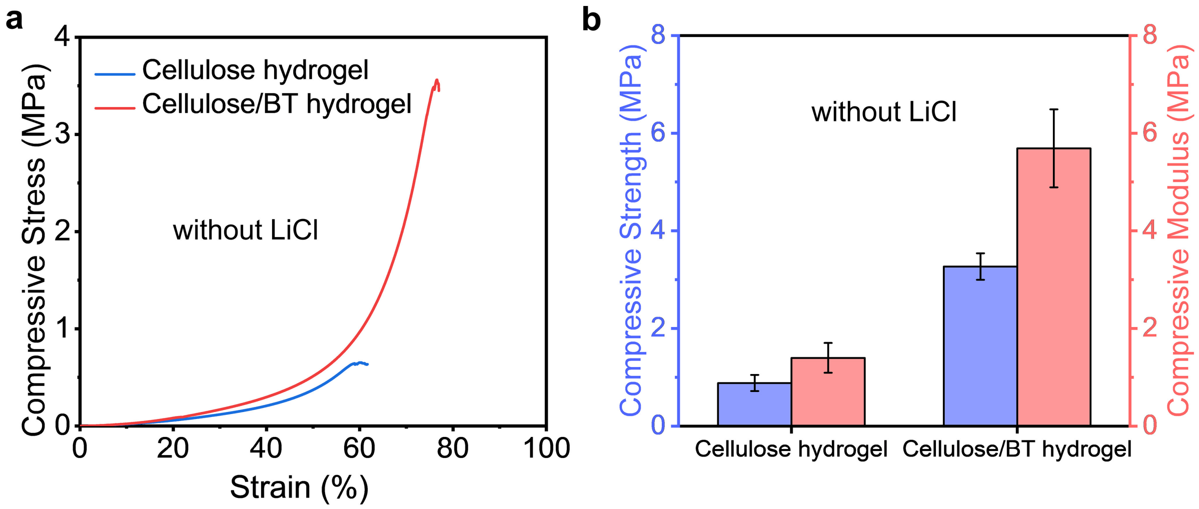

Supplementary Fig. 16| a Compressive stress-strain curves, **b** compressive strength and modulus of hydrogels without LiCl. Data are presented as mean values ± SD, $n = 3$ independent samples. Cellulose/BT hydrogels immersed in LiCl solution have good mechanical properties. The compressive strength and compressive modulus of the cellulose/BT hydrogel sample before and after soaking in LiCl solution are 3.56 MPa, 5.69 MPa, and 3.2 MPa, 5.65 MPa, respectively. In contrast, as LiCl weakens the hydrogen bonds between cellulose chains, the compressive strength and compressive modulus of the cellulose hydrogel sample decreased from 0.65 MPa, 1.4 MPa to 0.17 MPa, 0.47 MPa, respectively.

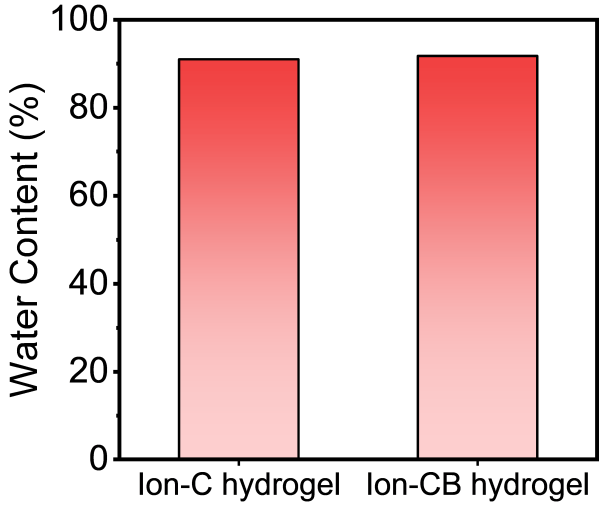

Supplementary Fig. 17 | The water content of hydrogels. The moisture contents of cellulose hydrogels are above 90%, indicating that nanocomposite hydrogels have high strength and high moisture content. To illustrate, the water contents of Ion-C hydrogel and Ion-CB hydrogel were 90.96% and 91.72%, respectively, whereas the compressive strength of Ion-CB hydrogel was indeed 18.9 times that of Ion-C hydrogel.

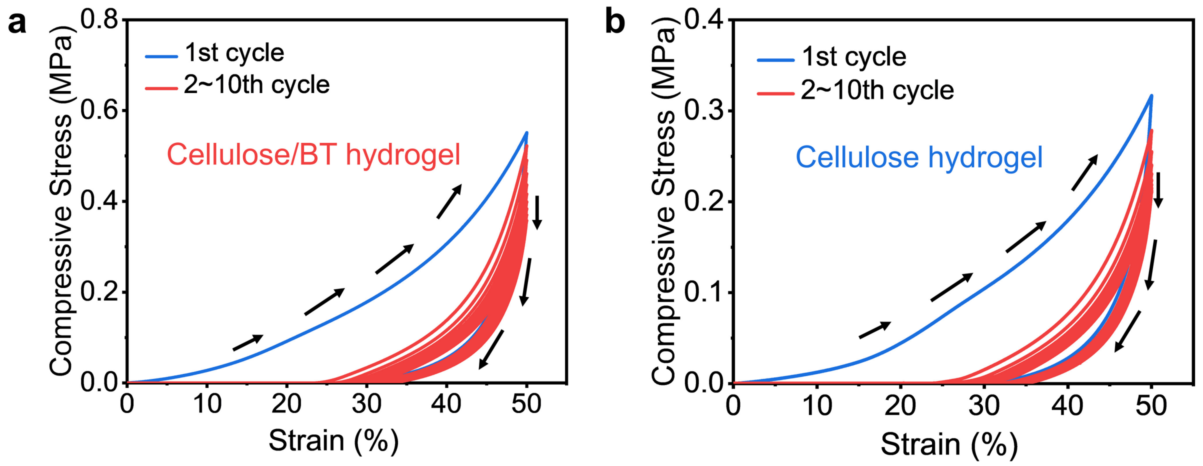

Supplementary Fig. 18 | a Cycle compressive stress-strain curves of the cellulose/hydrogel without LiCl at a maximum strain of 50%. **b** Cycle compressive stress-strain curves of the cellulose hydrogel without LiCl at a maximum strain of 50%. Compared to the cellulose hydrogel sample, the compressive strength of the cellulose/BT hydrogel sample at 50% compressive strain is higher, indicating that BT can improve the mechanical properties of the hydrogel.

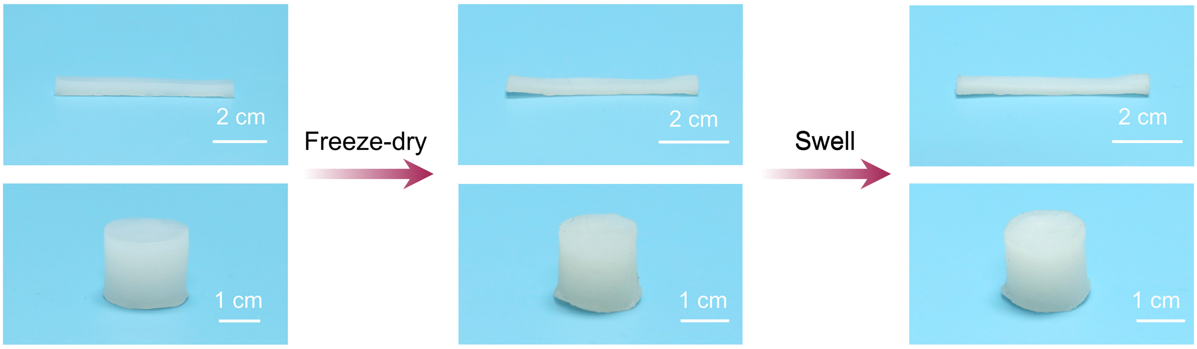

Supplementary Fig. 19 | Reswelling behavior of the freeze-dried Ion-CB hydrogel. No obvious dimension shrinkage was observed for the Ion-CB hydrogel after freeze-dried, which can nearly completely recover to its original shape after reswelling treatment.

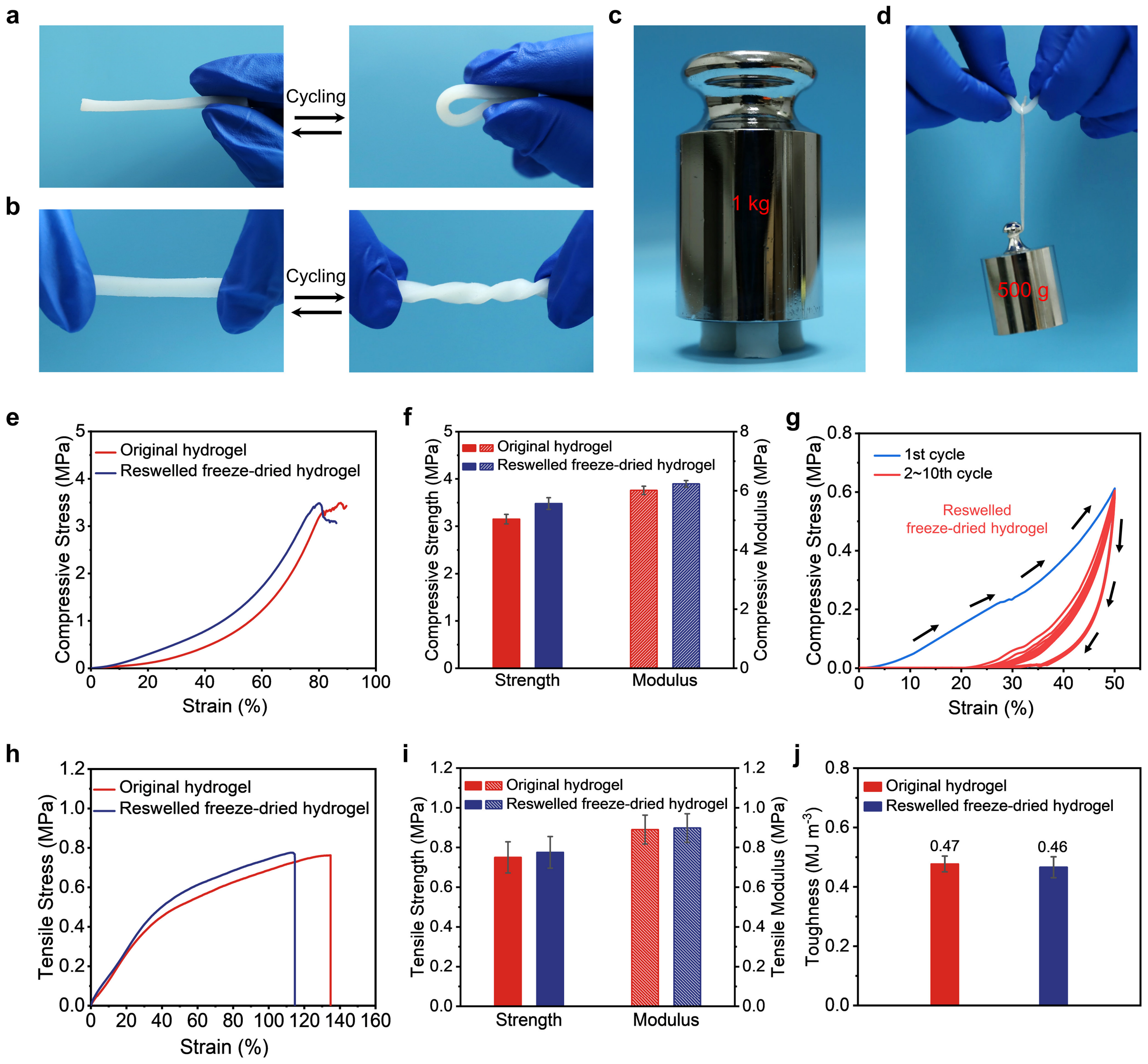

Supplementary Fig. 20 | Mechanical properties of the reswelled Ion-CB hydrogels. a-d Optical images of reswelled hydrogels after freeze-drying treatment (**a**) bending, (**b**) twisting, (**c**) holding, and (**d**) lifting under external forces. **e** Compressive stress-strain curves of the hydrogels. **f** Compressive strength and compressive modulus of the hydrogels. **g** Cyclic compressive stress-strain curves of the hydrogel with a maximum strain of 50%. **h** Tensile stress-strain curves of the hydrogels. **i** Tensile strength and tensile modulus of the hydrogels. **j** Toughness of the hydrogels. Data in (**f, i,** and **j**) are presented as mean values ± SD, n = 3 independent samples.

We further explored the mechanical properties of the reswelled hydrogels. As shown in the Supplementary Fig. 20a-d, cyclic bending and twisting tests reveal that the reswelled hydrogel can recover its initial shape immediately once the external force is removed, as an

indicator of good resilience. The reswelled hydrogel can easily hold a 1 kg and pull up a 500 g weight with minor deformation, evidencing its good mechanical robustness.

Compressive and tensile stress-strain tests were performed to quantitatively investigate the mechanical properties of the reswelled hydrogel samples. As shown in Supplementary Fig. 20e, the reswelled Ion-CB hydrogel after freeze-dried demonstrates a maximum compression stress of 3.5 MPa at a fracture strain of 80%, comparable to that of the original Ion-CB hydrogel. Meanwhile, the compressive modulus of the reswelled hydrogel after freeze-dried is similar to the original Ion-CB hydrogel (Supplementary Fig. 20f). Cyclic compressive tests at a fixed strain of 50% further showed that the reswelled hydrogel after freeze-dried maintained the excellent compressibility and strain memory effects of the original hydrogel (Supplementary Fig. 20g).

The tensile stress-strain curves show that the tensile fracture stress and modulus of the reswelled Ion-CB hydrogel after freeze-dried reached 0.77 and 0.90 MPa at a high fracture strain of 114%, respectively, similar to the original Ion-CB hydrogel (Supplementary Fig. 20h-i). In addition, the reswelled Ion-CB hydrogel after freeze-dried demonstrated an impressive high toughness of 0.46 MJ m^{-3} that is comparable to the original Ion-CB hydrogel (Supplementary Fig. 20j).

The above results indicated that the reswelled Ion-CB after freeze-dried shows no mechanical degradation.

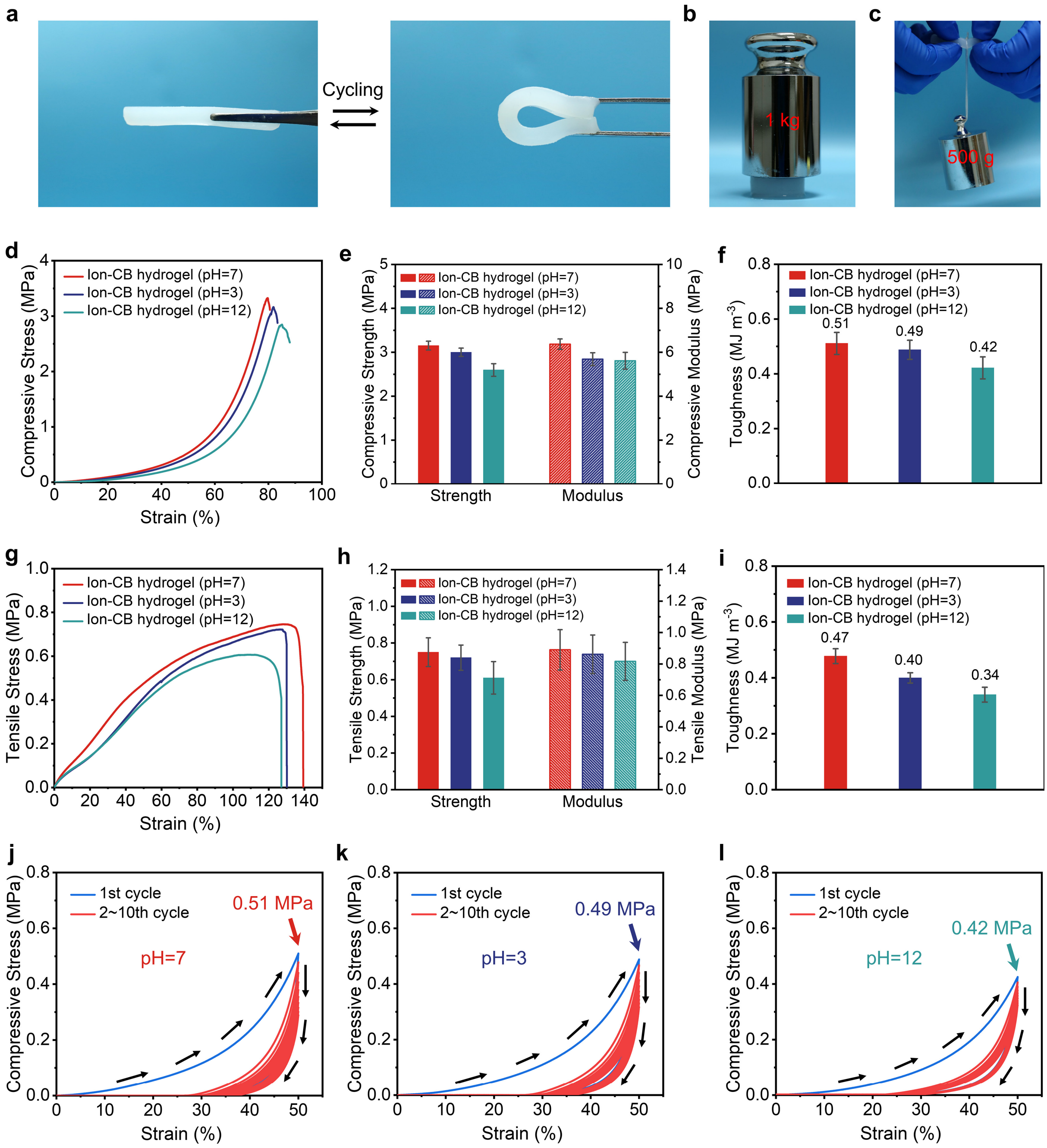

Supplementary Fig. 21 | Mechanical properties of Ion-CB hydrogels at under pH conditions. a Cyclic bending test of Ion-CB hydrogel after acid treatment. **b** Compressive test of the alkali-treated Ion-CB hydrogel under the external force. **c** Tensile test of the alkali-treated Ion-CB hydrogel under the external force. (**d**) Compressive stress-strain curves, (**e**) compressive strength and compressive modulus, and (**f**) compressive toughness of Ion-CB hydrogels at varied pH environments. (**g**) Tensile stress-strain curves, (**h**) tensile strength and tensile modulus, and (**i**) tensile toughness of Ion-CB hydrogels at varied pH environments. Cyclic compressive stress-strain curves of Ion-CB hydrogels in (**j**) neutral, (**k**) acidic, and (**l**)

alkaline environments with a maximum strain of 50%. Data in (**e, f, h,** and **i**) are presented as mean values ± SD, $n = 3$ independent samples.

Supplementary Fig. 21a shows the cyclic bending test of the hydrogel, even under an acidic condition, the Ion-CB hydrogel can recover its initial shape immediately after removing the external force, indicating good resilience. As shown in Supplementary Fig. 21b-c, the Ion-CB hydrogel that is treated by a strong alkaline environment can easily hold a 1 kg and lift 500 g weight with minor deformation, which is an indicator of high stiffness and toughness.

Compression and tensile stress-strain tests were performed to explore the mechanical properties of hydrogel samples. As shown in Supplementary Fig. 21d, compared with the neutral Ion-CB hydrogel, the maximum compressive stress of the hydrogel after acid/alkali treatment is slightly reduced, suggesting their excellent mechanical compressive performance under neutral/acidic/alkaline environments. Slight decrease in the compressive modulus and toughness of the hydrogel after acid/alkali treatment was also observed (Supplementary Fig. 21e-f), suggesting that neutral environment is the best condition. Similarly, the tensile test results showed that the tensile strength, modulus and toughness of the Ion-CB hydrogels under acidic and alkaline environments decreased slightly compared with the neutral Ion-CB hydrogel (Supplementary Fig. 21g-i). Among them, the alkali-treated Ion-CB hydrogels showed the lowest toughness of 0.34 MJ m^{-3}, suggesting that alkaline environment is not preferred for fabricating mechanically strong Ion-CB hydrogel.

Cyclic compression tests at a fixed strain of 50% were conducted to further explore the effect of pH on the mechanical strength of Ion-CB hydrogels. As displayed in Supplementary Fig. 21j-l, Ion-CB hydrogels in all environments exhibit nonlinear elastic-inelastic behavior, characterized by large hysteresis and shape recovery upon unloading in the 1st cycle, indicating that energy dissipation during the deformation process is mainly ascribed to the destruction of hydrogen bonds.

Expectantly, for all Ion-CB hydrogels, each loading curve can reach the peak value of the 1st cycle, suggesting that the strong coordination network can well prevent the fracture during the deformation process. Spontaneously, nearly overlapped curves are observed from the 2nd to

10th cycles, which reveals a splendid strain memory effect. Moreover, compared with the neutral Ion-CB hydrogel, the compressive stress at a strain of 50% is 0.51 MPa, and the compressive stress under the corresponding strain of the hydrogel after acid and alkali treatment is 0.49 and 0.42 MPa, respectively, which still maintains competitive advantage.

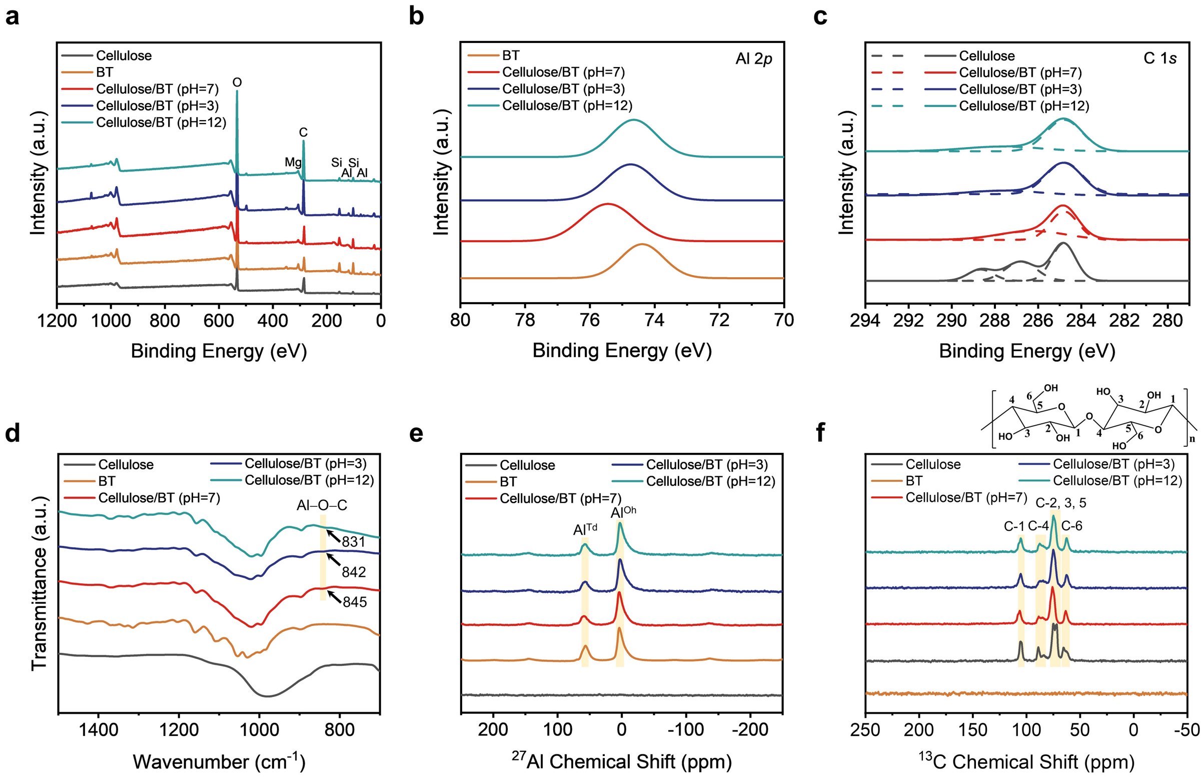

Supplementary Fig. 22 | Structure analysis of Ion-CB hydrogels under varied pH conditions. a XPS spectra of cellulose, BT, and Ion-CB hydrogels under varied pH conditions. **b** Al 2*p* orbital XPS spectra of BT and Ion-CB hydrogels under varied pH conditions**. c** C 1*s* orbital XPS spectra of cellulose and Ion-CB hydrogels under varied pH conditions. **d** FTIR spectra of cellulose, BT, and Ion-CB hydrogels under varied pH conditions. (**e**) ^{27}Al and (**f**) ^{13}C solid-state MAS NMR spectra of cellulose, BT, and Ion-CB hydrogels under varied pH conditions.

As shown in Supplementary Fig. 22a, the appearance of characteristic peaks of the XPS spectra indicates that BT stably exists in the Ion-CB hydrogels even in harsh (acidic or alkaline) environments. Furthermore, by comparing XPS spectra obtained from BT and the Ion-CB hydrogel, the characteristic peak of Al 2*p* shifts from 74.4 eV for BT to 75.2 eV for the Ion-CB hydrogel in neutral environment, which is an indicator of the formation of Al–O–C bond (Supplementary Fig. 22b). When the Ion-CB hydrogel was exposed to an extreme environment (acidic and alkaline environments), the peak slightly shifts to a lower binding energy (74.7 for acidic environment and 74.6 eV for alkaline environment, both are still higher than 74.4 eV of BT), indicating the weakening of Al–O–C bond. The C 1*s* spectrum of cellulose shows three

deconvoluted peaks at 284.8, 286.2, and 288.7 eV, which are attributed to $–C–H_2$, –C–O–H, and –O–C–O/–C=O bonds, respectively, and the intensity of the –C–O–H, and –O–C–O/–C=O bonds appear to decrease significantly due to the interaction of cellulose with BT in the formed Ion-CB hydrogel (Supplementary Fig. 22c)[3].

Through the observation of FTIR (Supplementary Fig. 22d), the Ion-CB hydrogel exhibits a prominent Al–O–C new peak at 845 cm^{-1} due to the strong interaction between cellulose and BT[4, 5]. When treated with acidic or alkaline solution, the characteristic peaks of the Ion-CB hydrogel red-shift slightly (842 cm^{-1} when pH = 3 and 831 cm^{-1} when pH = 12), suggesting the slight weakening of the Al–O–C bond in acidic or alkaline environments.

The MAS NMR spectra of ^{27}Al from Supplementary Fig. 22e show two signals in all samples. For the BT platelets, the peak at 57.5 ppm corresponds to the tetrahedrally coordinated aluminum (in the lattice cell of $[AlO_4]$, donated as Al^{Td}) and the second signal at 3.5 ppm to the aluminum in an octahedral position (in the lattice cell of $[AlO_6]$, donated as Al^{Oh}). The chemical shift of the characteristic peaks in the ^{27}Al MAS NMR spectra of the three Ion-CB hydrogels under neutral/acidic/alkaline environments remains unchanged compared to that of BT, indicating the coordination environment of aluminum in the BT platelets and the Ion-CB hydrogels with varied pH values still only exists the Al^{Td} and Al^{Oh} (refs 3, 6). However, compared with the stronger Al^{Td} signal in the BT platelets, the Al^{Td} signal intensities in three Ion-CB hydrogels decrease, which is due to the formation of Al^{Oh} when cellulose coordinates with the Al^{Td} on the BT surface[7]. The Al^{Oh} signal intensity of the BT spectrum is 1.85 times higher than that of Al^{Td}, calculated from the integral areas of Al^{Oh} and Al^{Td} peaks, while the corresponding value is 3.52 times in the Ion-CB hydrogel, an improvement of about 85% compared to BT platelets was observed, indicating a strong interaction between and BT. When treated with acidic or alkaline solutions, the area ratio of Al^{Oh} to Al^{Td} decreases slightly (3.34 times when pH = 3 and 3.16 times when pH = 12), suggesting that the Al–O–C bond are only slightly broken in acidic or alkaline environments. For the MAS NMR spectra of ^{13}C displayed in Supplementary Fig. 22f, chemical shifts demonstrate several characteristic peaks of cellulose: peaks at 71.4 and 74.9 ppm are associated with the C-2, C-3, and C-5, peak at 65.0 ppm ascribes

to C-6, peak at 88.9 ppm corresponds to C-4, and peak at 104.3 ppm belongs to C-1. Obviously, although no new peaks appear for the three Ion-CB hydrogels, the sharpness of the peaks decreases, attributed to the increase of amorphous region (or the decrease of crystallinity) of cellulose. At the same time, compared with the C-6 peak of cellulose, the Ion-CB hydrogel has a higher magnetic field, indicating that the intramolecular hydrogen bonds in cellulose are destroyed, and the crystal structure of cellulose shifts from cellulose I to cellulose II, which is consistent with the literature[1, 2].

The above results all point to the formation of Al–O–C bond between the hydroxyls from cellulose and the Al units on BT.

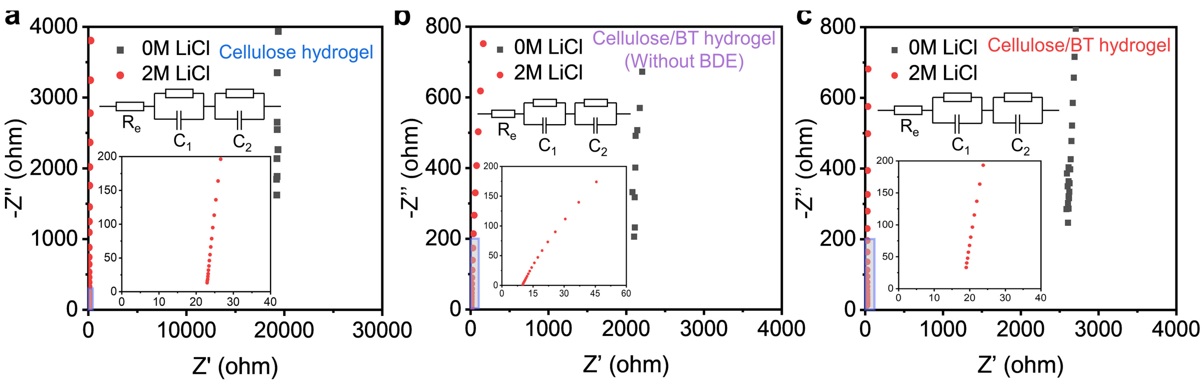

Supplementary Fig. 23 | EIS Nyquist plot of (**a**) cellulose hydrogel, (**b**) cellulose/BT hydrogel without BDE, and (**c**) cellulose/BT hydrogel after soaking in 0 and 2 M LiCl solution. As the concentration of LiCl increases, the resistance decreases, indicating that the ionic conductivity of the hydrogel is higher.

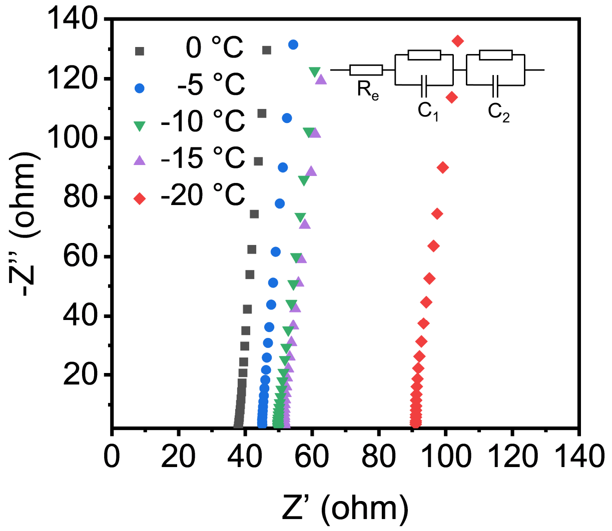

Supplementary Fig. 24 | EIS Nyquist plot of cellulose/BT hydrogel immersed in 2 M LiCl solution at 0, −5, −10, −15, and −20 °C. As the temperature decreases, the resistance increases, indicating that the ionic conductivity of the hydrogel decreases.

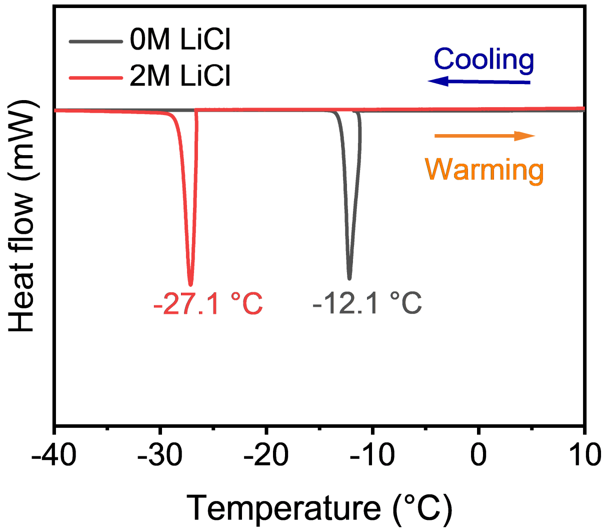

Supplementary Fig. 25 | DSC curves of cellulose hydrogel without soaking and immersing in 2 M LiCl solution. After the hydrogel was immersed in 2 M LiCl, the freezing point decreased from −12.1 to −27.1 °C, which was due to the formation of a salt/water system in the hydrogel.

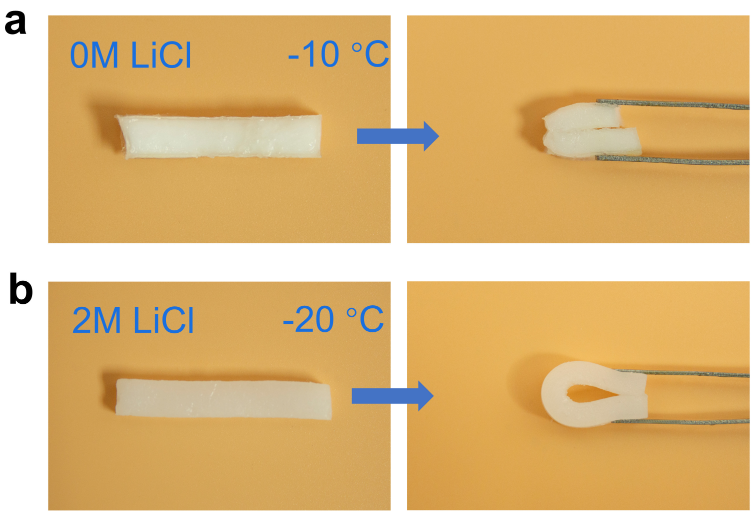

Supplementary Fig. 26 | a Photographs of the cellulose/BT hydrogel without LiCl treatment after freezing at −10 °C. **b** Photographs of the cellulose/BT within LiCl treatment after freezing at −20 °C. When the temperature dropped to −10 °C, cellulose/BT hydrogel samples that are not immersed in LiCl solution turned into white opaque color and were prone to breakage under external force, which is due to the formation of ice crystals inside the hydrogel. In contrast, the hydrogel sample after diffusion treatment in 2 M LiCl solution still maintained certain transparency, soft, elastic, and flexible even under −20 °C. Such impressive icing tolerant behavior can be attributed to the salt/H_2O solvent system in hydrogel networks.

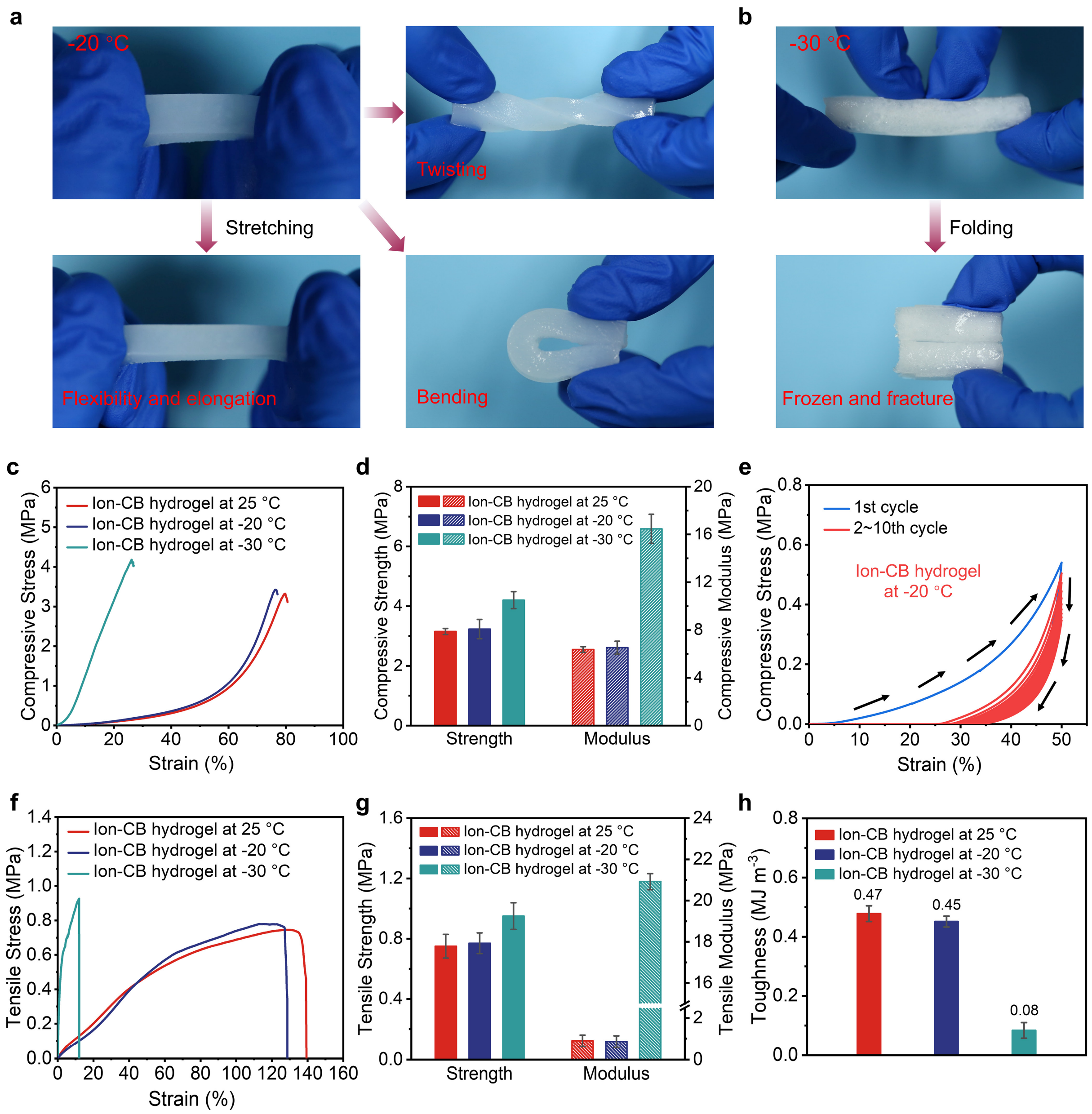

Supplementary Fig. 27 | Mechanical properties of Ion-CB hydrogel at low temperature. Optical images of Ion-CB hydrogels under external forces at (**a**) −20 and (**b**) −30 °C, respectively. **c** Compressive stress-strain curves of the Ion-CB hydrogels at varied temperature. **d** Compressive strength and compressive modulus of the Ion-CB hydrogels at varied temperature. **e** Cyclic compressive stress-strain curves of the Ion-CB hydrogels with a maximum strain of 50% at −20 °C. **f** Tensile stress-strain curves of the Ion-CB hydrogels at varied temperature. **g** Tensile strength and tensile modulus of Ion-CB hydrogels at varied temperature. **h** Toughness of the Ion-CB hydrogels at varied temperature. Data in (**d, g,** and **h**) are presented as mean values ± SD, *n* = 3 independent samples.

As shown in Supplementary Fig. 27a, the Ion-CB hydrogel maintains a certain transparency and can be easily stretched, twisted and bent, demonstrating excellent softness, elasticity and flexibility even at −20 °C. Such impressive icing tolerant behavior is attributed to the salt/H_2O solvent system in the hydrogel networks. However, when the temperature dropped to −30 °C, the Ion-CB hydrogel turned into white opaque color and was prone to fracture under the external force, which is due to the formation of ice crystals inside the hydrogel (Supplementary Fig. 27b). Such phenomenon is rational as according to the DSC measurement result in Fig. 5d in the revised manuscript, the freezing point of the Ion-CB hydrogel is −28.9 °C, so the hydrogel freezes inevitably at −30 °C.

As shown in Supplementary Fig. 27c, the maximum compressive stresses of the Ion-CB hydrogels at 25 and −20 °C are 3.3 MPa at fracture strain of 80% and 3.4 MPa at fracture strain of 77%, respectively, exhibiting no significant difference. However, due to the freezing of the hydrogel at −30 °C, the maximum compressive stress is 4.2 MPa at fracture strain of 26% and the compressive modulus increases by 2.5 times. The compressive modulus of the hydrogel at −20 °C is also comparable to the Ion-CB hydrogel at 25 °C (Supplementary Fig. 27d). Cyclic compressive tests at a fixed strain of 50% from Supplementary Fig. 27e further showed that the Ion-CB hydrogel at −20 °C had excellent compressibility and strain memory effects.

The tensile stress-strain curves demonstrate that the tensile fracture stress and modulus of the Ion-CB hydrogel at −20 °C reached 0.77 and 0.85 MPa at a high fracture strain of 123%, respectively, showing no significant changes compared with the Ion-CB hydrogel at 25 °C (Supplementary Fig. 27f-g). In addition, the Ion-CB hydrogel still has an impressive high fracture energy of 0.45 MJ m^{-3} even at −20 °C, while the toughness of the hydrogel at −30 °C is greatly weakened by freezing (Supplementary Fig. 27h). Therefore, the Ion-CB hydrogel developed in this work possesses excellent mechanical properties even at an extremely low environmental temperature of −20 °C, which promises the great potential of our all-natural hydrogel for practical applications.

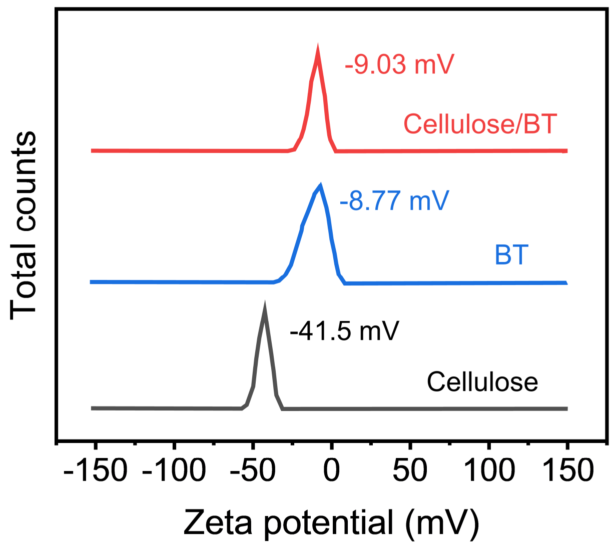

Supplementary Fig. 28 | Zeta potential of cellulose, BT, and cellulose/BT slurry. The Zeta potential of the cellulose/BT slurry is –9.03 mV, which can attract positive ions to move between the BT layers.

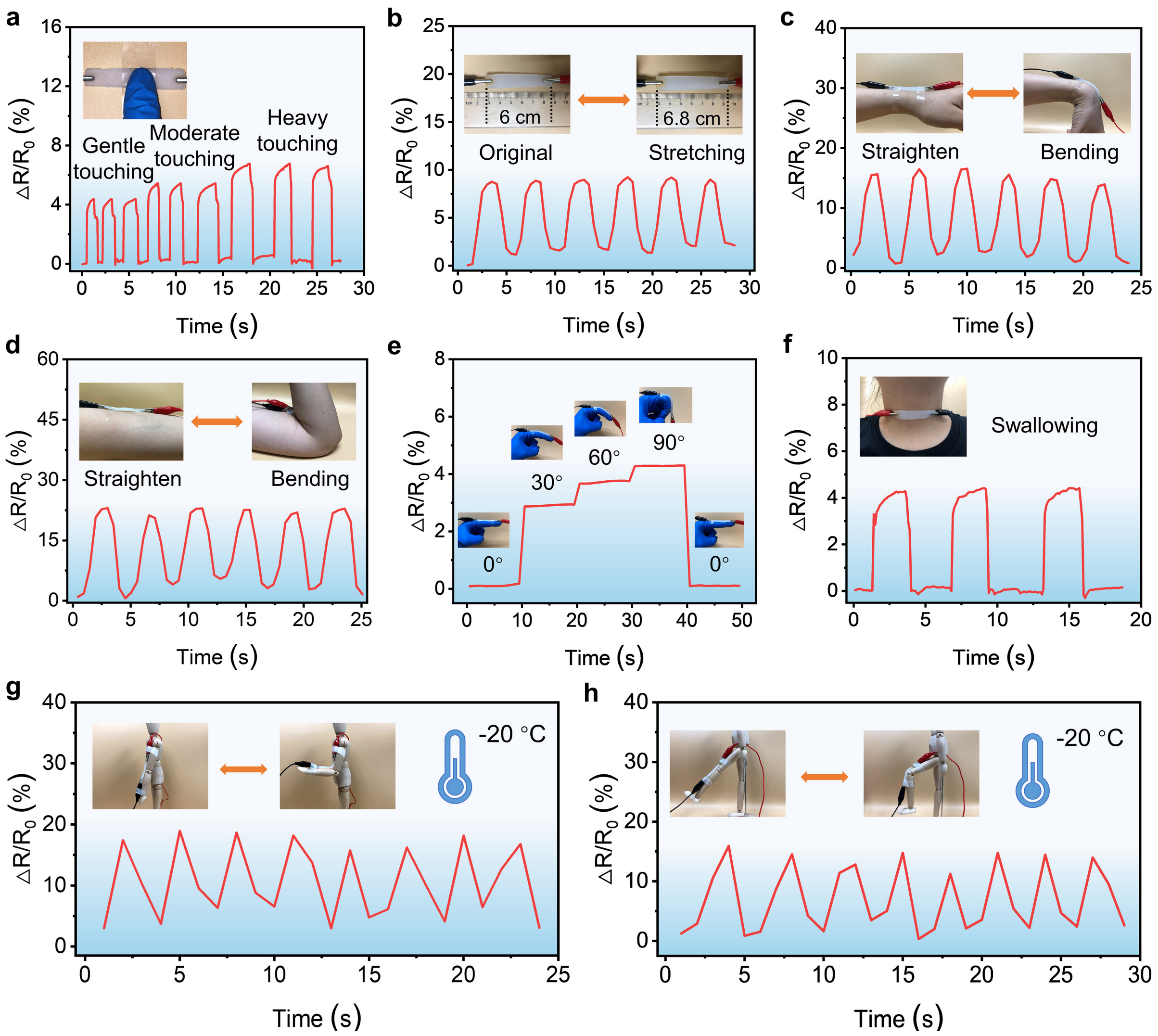

Supplementary Fig. 29 | Advanced Ion-CB hydrogel-based sensor in monitoring human joints and physiological signals. The relative resistance changes of the hydrogel sensor during (**a**) touching and (**b**) stretching processes. The recorded relative resistance changes of the hydrogel sensor in response to bending and releasing of (**c**) wrist, (**d**) elbow, (**e**) finger, and (**f**) swallowing. The hydrogel sensors attached to a human model for detecting (**g**) elbow and (**h**) knee rotation at −20 °C. At room temperature and −20 °C, the resistance of the Ion-CB hydrogel can show repeated and stable responsive changes during compression and bending and can be used as a flexible sensor to monitor body movement and physiological signals. The peak part of the relative resistance changes curves indicates that the hydrogel is deformed, and the valley part shows it returned to its original state.

Supplementary Table 1 | Summary of ionic conductive and anti-freezing hydrogels.

References	Conductive components	Anti-freezing agents	Freezing point (°C)	Conductivity at room temperature (mS/cm)	Conductivity at −20 °C (mS/cm)
**This work**	**2M LiCl (soaking)**	**2M LiCl (soaking)**	**−28.9**	**89.9**	**25.8**
[8]	30wt% $CaCl_2$ (soaking)	30wt% $CaCl_2$ (soaking)	−57	≈80.0	≈2.0
[9]	2M $ZnSO_4$ (loading)	Ethylene glycol (loading)	−20	16.8	14.6
[10]	Polyaniline (loading)	Glycerol (loading)	−20	≈23.0	4.5
[11]	3wt% LiCl (loading)	3wt% LiCl (loading)	−21	75.3	N/A
[12]	4wt% LiCl (loading)	3wt% Ethylene glycol (loading)	−40	≈16.0	2.38
[13]	1M H_2SO_4 (loading)	Ethylene glycol (loading)	−40	≈30.0	4.8
[14]	5M KOH (soaking)	5M KOH (soaking)	−20	105.0	18.1
[15]	2M $ZnSO_4$+0.2M $MnSO_4$ (loading)	Glycerol (loading)	−35	96.3	10.1
[16]	2M H_2SO_4 (soaking)	DMSO (soaking)	−50	≈7.0×10^{-2}	1.7×10^{-2}
[17]	MXene (loading)	Ethylene glycol (soaking)	−40	≈0.18	≈2.0×10^{-2}
[18]	2M $BzMe_3NOH$ (loading)	2M $BzMe_3NOH$ (loading)	−24	23.7	N/A
[19]	1.1%$FeCl_3$+2M NaCl (soaking)	1.1%$FeCl_3$+2M NaCl (soaking)	−24	7.2	N/A
[20]	4M LiCl+2M $ZnSO_4$	4M LiCl+2M $ZnSO_4$	−20	≈34.7	≈10.4
[21]	$ZnCl_2$ (loading)	$ZnCl_2$ (loading)	−20	74.9	47.7

Supplementary Table 2 | Formulation of different cellulose/BT hydrogels.

Sample	Cellulose (g)	Alkaline solution (g)	Bentonite (g)	LiCl concentration (mol/L)
Cellulose hydrogel	3.0	97.0	0.00	0
Cellulose/BT hydrogel	3.0	97.0	0.30	0
Ion-C hydrogel	3.0	97.0	0.00	2
Ion-CB hydrogel	3.0	97.0	0.30	2

Supplementary References

1. Zhang, L., Ruan, D. & Gao, S. Dissolution and regeneration of cellulose in NaOH/thiourea aqueous solution. *J. Polym. Sci, Part B: Polym. Phys.* **40**, 1521-1529 (2002).
2. Cai, J. & Zhang, L. Rapid dissolution of cellulose in LiOH/urea and NaOH/urea aqueous solutions. *Macromol. Biosci.* **5**, 539-548 (2005).
3. Podsiadlo, P. et al. Ultrastrong and stiff layered polymer nanocomposites. *Science* **318**, 80-83 (2007).
4. Wang, J., Cheng, Q., Lin, L. & Jiang, L. Synergistic toughening of bioinspired poly(vinyl alcohol)-clay-nanofibrillar cellulose artificial nacre. *ACS Nano* **8**, 2739-2745 (2014).
5. Chen, G. et al. A strong, flame-retardant, and thermally insulating wood laminate. *Chem. Eng. J.* **383**, 123109 (2020).
6. Pentrák, M. et al. Alteration of fine fraction of bentonite from Kopernica (Slovakia) under acid treatment: a combined XRD, FTIR, MAS NMR and AES study. *Appl. Clay Sci.* **163**, 204-213 (2018).
7. Podsiadlo, P., Shim, B. S. & Kotov, N. A. Polymer/clay and polymer/carbon nanotube hybrid organic–inorganic multilayered composites made by sequential layering of nanometer scale films. *Coord. Chem. Rev.* **253**, 2835-2851 (2009).
8. Morelle, X. P. et al. Highly stretchable and tough hydrogels below water freezing temperature. *Adv. Mater.* **30**, 1801541 (2018).
9. Mo, F. et al. A flexible rechargeable aqueous zinc manganese-dioxide battery working at −20 °C. *Energy Environ. Sci.* **12**, 706-715 (2019).
10. Shang, Y., Wei, J., Wu, C. & Wang, Q. Extreme temperature-tolerant organohydrogel electrolytes for laminated assembly of biaxially stretchable pseudocapacitors. *ACS Appl. Mater. Interfaces* **10**, 42959-42966 (2018).
11. Guan, L., Yan, S., Liu, X., Li, X. & Gao, G. Wearable strain sensors based on casein-driven tough, adhesive and anti-freezing hydrogels for monitoring human-motion. *J. Mater. Chem. B* **7**, 5230-5236 (2019).
12. Rong, Q., Lei, W., Huang, J. & Liu, M. Low temperature tolerant organohydrogel electrolytes for flexible solid-state supercapacitors. *Adv. Energy Mater.* **8**, 1801967 (2018).

13. Liu, Z. et al. Highly compressible and superior low temperature tolerant supercapacitors based on dual chemically crosslinked PVA hydrogel electrolytes. *J. Mater. Chem. A* **8**, 6219-6228 (2020).
14. Sun, N. et al. Alkaline double-network hydrogels with high conductivities, superior mechanical performances, and antifreezing properties for solid-state zinc-air batteries. *ACS Appl. Mater. Interfaces* **12**, 11778-11788 (2020).
15. Chen, M. et al. Anti-freezing flexible aqueous Zn–MnO_2 batteries working at −35 °C enabled by a borax-crosslinked polyvinyl alcohol/glycerol gel electrolyte. *J. Mater. Chem. A* **8**, 6828-6841 (2020).
16. Lu, C. & Chen, X. All-temperature flexible supercapacitors enabled by antifreezing and thermally stable hydrogel electrolyte. *Nano Lett.* **20**, 1907-1914 (2020).
17. Liao, H., Guo, X., Wan, P. & Yu, G. Conductive MXene nanocomposite organohydrogel for flexible, healable, low - temperature tolerant strain sensors. *Adv. Funct. Mater.* **29**, 1904507 (2019).
18. Wang, Y., Zhang, L. & Lu, A. Transparent, antifreezing, ionic conductive cellulose hydrogel with stable sensitivity at subzero temperature. *ACS Appl. Mater. Interfaces* **11**, 41710-41716 (2019).
19. Li, S., Pan, H., Wang, Y. & Sun, J. Polyelectrolyte complex-based self-healing, fatigue-resistant and anti-freezing hydrogels as highly sensitive ionic skins. *J. Mater. Chem. A* **8**, 3667-3675 (2020).
20. Zhu, M. et al. Antifreezing hydrogel with high zinc reversibility for flexible and durable aqueous batteries by cooperative hydrated cations. *Adv. Funct. Mater.* **30**, 1907218 (2019).
21. Yang, L. et al. Zinc ion trapping in a cellulose hydrogel as a solid electrolyte for a safe and flexible supercapacitor. *J. Mater. Chem. A* **8**, 12314-12318 (2020).
