# Supplementary material for: Strong, tough, ionic conductive, and freezing-tolerant all-natural hydrogel enabled by cellulose-bentonite coordination interactions
Source: Nat Commun. 2022 Jun 21;13:3408. doi: 10.1038/s41467-022-30224-8 (PMC9213515; doi:10.1038/s41467-022-30224-8)
Supplement: Supplementary file 2 — Description of Additional Supplementary Files [file 41467_2022_30224_MOESM2_ESM.pdf]

Description of Additional Supplementary Files

Supplementary Movie 1: Mechanical properties of the Ion-CB hydrogel. The Ion-CB hydrogel is fabricated into spherical shapes that can be hit multiple times as a ping pong ball without cracking, indicating its impressive mechanical flexibility and durability.

Supplementary Movie 2: Ionic conductive properties of the Ion-CB hydrogel. A light-emitting diode (LED) can be lighted cyclically with a easy capacity when the Ion-CB hydrogel acts as an ionic conductor, proving it possesses excellent ionic conductivity.

Supplementary Movie 3: Sensing properties of the Ion-CB hydrogel. when the Ion-CB hydrogel is adhered to the human model and bent at –20 °C, repeatable resistance responses with a high signal-to-noise ratio are also obtained, demonstrating a long service life and good reliability of hydrogel.
